# A dual-specific macrophage colony-stimulating factor antagonist of c-FMS and α_v_β_3_ integrin for osteoporosis therapy

**DOI:** 10.1371/journal.pbio.2002979

**Published:** 2018-08-24

**Authors:** Yuval Zur, Lior Rosenfeld, Chen Anna Keshelman, Nofar Dalal, Gali Guterman-Ram, Ayelet Orenbuch, Yulia Einav, Noam Levaot, Niv Papo

**Affiliations:** 1 Department of Biotechnology Engineering and the National Institute of Biotechnology in the Negev, Ben-Gurion University of the Negev, Beer-Sheva, Israel; 2 The National Institute for Biotechnology in the Negev (NIBN), Beer-Sheva, Israel; 3 Department of Physiology and Cell Biology, Regenerative Medicine and Stem Cell Research Center (RMSC), Ben-Gurion University of the Negev, Beer-Sheva, Israel; 4 Faculty of Engineering, Holon Institute of Technology, Holon, Israel; University of London, The Royal Veterinary College, United Kingdom of Great Britain and Northern Ireland

## Abstract

There is currently a demand for new highly efficient and specific drugs to treat osteoporosis, a chronic bone disease affecting millions of people worldwide. We have developed a combinatorial strategy for engineering bispecific inhibitors that simultaneously target the unique combination of c-FMS and α_v_β_3_ integrin, which act in concert to facilitate bone resorption by osteoclasts. Using functional fluorescence-activated cell sorting (FACS)-based screening assays of random mutagenesis macrophage colony-stimulating factor (M-CSF) libraries against c-FMS and α_v_β_3_ integrin, we engineered dual-specific M-CSF mutants with high affinity to both receptors. These bispecific mutants act as functional antagonists of c-FMS and α_v_β_3_ integrin activation and hence of osteoclast differentiation in vitro and osteoclast activity *in vivo*. This study thus introduces a versatile platform for the creation of new-generation therapeutics with high efficacy and specificity for osteoporosis and other bone diseases. It also provides new tools for studying molecular mechanisms and the cell signaling pathways that mediate osteoclast differentiation and function.

## Author summary

Many bone diseases—including osteoporosis, in which the bones become brittle and fragile from loss of tissue—are characterized by excessive and uncontrolled bone resorption by bone-destroying cells known as osteoclasts. Therefore, controlled and specific inhibition of osteoclast activity is a desired outcome in treatments for bone diseases. Osteoclast differentiation and function are coordinated by cell surface receptors, including c-FMS and α_v_β_3_ integrin, which cooperate with one another to drive signals that are essential for osteoclast functions. Here, we describe the engineering, characterization, and testing of novel proteins that can target and inhibit both c-FMS and α_v_β_3_ integrin at the same time, thereby providing a way of controlling osteoclast function. The study represents the first example of engineering a natural ligand, which acts as a signaling molecule, as a scaffold for binding not only its target protein but also a second target. We show that these engineered proteins inhibit osteoclast activity in a mouse model of osteoporosis. Our study describes potential inhibitors that target all the known functions resulting from c-FMS/integrin α_v_β_3_ crosstalk and paves the way to create novel targeting proteins that could be used to treat osteoporosis. It also expands our understanding of the role of the c-FMS/α_v_β_3_ integrin pathway in the regulation of osteoclast differentiation and function.

## Introduction

Osteoporosis, a chronic skeletal disorder common in both women and men beyond the age of 50 [1], is the underlying cause of more than 8.9 million fractures annually worldwide, with the consequent high burden of social and economic costs [2]. The drugs initially used for the management of osteoporosis in women were based on estrogens, but long-term administration of these drugs is associated with increased risk of breast cancer, cardiovascular disease, and dementia [3–5]. To date, the most commonly prescribed drugs for osteoporosis are bisphosphonates, but these drugs, too, have nonspecific adverse side effects, such as gastrointestinal toxicity, renal toxicity, hypercalcemia, osteonecrosis of the jaw, and more [6]. A different therapeutic direction was recently opened with the Food and Drug Administration (FDA) approval of the receptor for activation of the nuclear factor–kappa B (NF-кB) ligand (RANKL) antibody, denosumab, but the clinical promise of this drug has been offset by reports of adverse side effects, such as hypocalcemia [7] and atypical hip fractures [8]. Thus, there is a pressing need for new efficient and highly specific drugs for the management of osteoporosis.

Central to the pathogenesis of osteoporosis is excessive bone resorption by osteoclasts [9]. These cells differentiate from cells of the monocyte/macrophage lineage upon stimulation of two essential factors, monocyte/macrophage colony-stimulating factor (M-CSF) and RANKL [10]. The importance of M-CSF and its receptor c-FMS in osteoclast function has been clearly illustrated in a study showing that both M-CSF–deficient and c-FMS–deficient mice suffer from retarded skeletal growth and osteopetrosis [11]. Another factor that is essential for osteoclast functioning is α_v_β_3_ integrin, as indicated, for example, in studies showing increased bone mass in integrin β_3_ knockout mice due to a functional defect in their osteoclasts [12–15]. The interaction of α_v_β_3_ integrin with the bone matrix induces a cytoskeleton organization that polarizes the osteoclast's resorptive machinery to the bone/cell interface, where it creates an isolated compartment consisting of an actin ring surrounding a ruffled border essential for resorption by the matured osteoclasts.

Importantly, in addition to the distinctive roles of c-FMS and α_v_β_3_ integrin in osteoclast activity, these two factors also play a cooperative role in bone resorption. M-CSF signaling regulates bone resorption by crosstalk through its receptor c-FMS, with the signaling being activated by α_v_β_3_ integrin. This signaling regulates cytoskeleton rearrangement, as both M-CSF and α_v_β_3_ integrin stimulate the same c-Src–initiated signaling complex essential for regulation of the osteoclast cytoskeleton [16–18]. In addition, c-FMS alters the conformation of α_v_β_3_ integrin from a low-affinity to a high-affinity state [19], and it activates the Rho GTPase family member Rac (which is essential for osteoclast cytoskeleton organization) in an α_v_β_3_-integrin–dependent manner [16, 20]. Moreover, it has been demonstrated that β_3_-integrin–deficient mice have elevated concentrations of serum M-CSF and that impaired expression of differentiation markers of β_3_-integrin–deficient osteoclasts can be rescued by an increased concentration of M-CSF, suggesting a compensation mechanism between the actions of these receptors [16]. In addition, c-FMS and α_v_β_3_ integrin colocalize to the osteoclast cytoskeleton, suggesting that the crosstalk between these receptors takes place at the same cellular sites. Indeed, it has been shown that an endogenic complex of c-FMS and α_v_β_3_ integrin coprecipitates from differentiated osteoclasts [21]. Thus, the functional and spatial coupling of c-FMS and α_v_β_3_ integrin in osteoclast differentiation and function, together with the osteoclast-exclusive presentation and crosstalk of these two receptors, suggests that an antagonist that could bind simultaneously to these two target receptors would serve as a highly specific and effective antiresorptive drug [19, 22].

**Fig 1:**
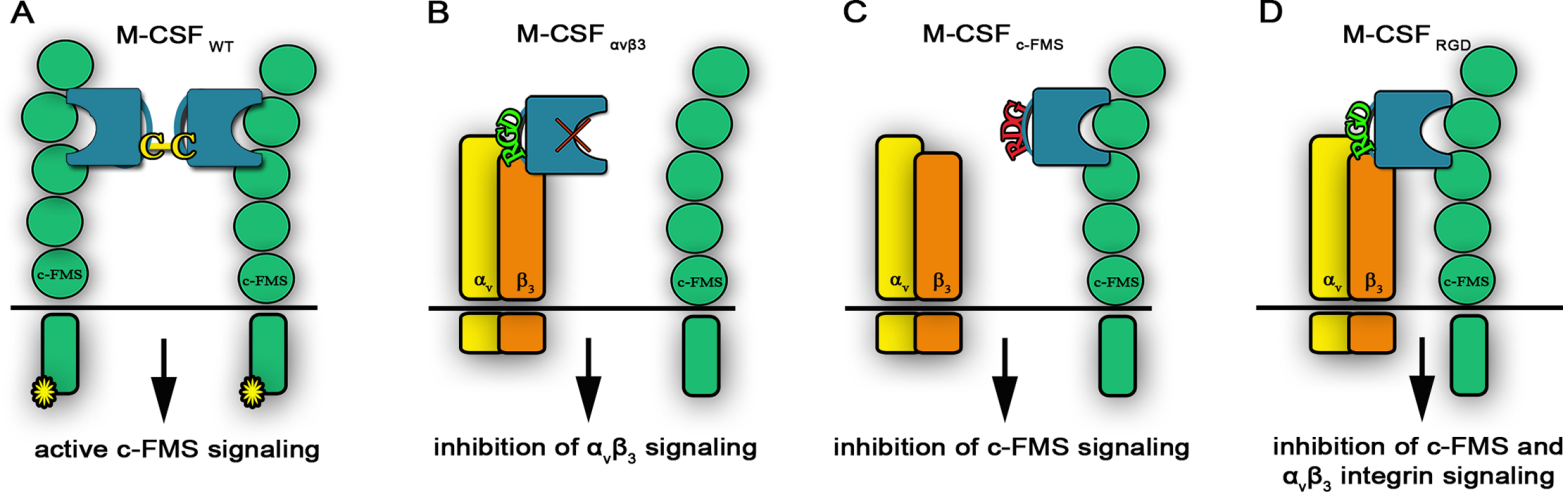
Schematic representation of the different M-CSF constructs. (A) WT M-CSF (designated M-CSF_WT_) constituting a disulfide-bond–linked homodimer. (B) Monospecific M-CSF that can bind α_v_β_3_ integrin (designated M-CSF_αvβ3_) via an RGD motif but not c-FMS because of mutations in positions 9 and 15. (C) Monospecific M-CSF that can bind only c-FMS (designated M-CSF_c-FMS_) because of two point mutations that change the RGD motif to RDG, thereby preventing its binding to α_v_β_3_ integrin. (D) Libraries (designated M-CSF_RGD_) created by changing two loops in the M-CSF dimerization site to an RGD motif with three random amino acids on each side, thereby enabling binding to α_v_β_3_ integrin. M-CSF, macrophage colony-stimulating factor; RGD, Arginine-Glycine-Aspartic acid; WT, wild type.

We thus aimed to exploit both the osteoclast's need for functional c-FMS and α_v_β_3_ integrin and the specific expression of these two factors as a platform for developing a new generation of bispecific c-FMS/α_v_β_3_ integrin antagonists for application in osteoporosis therapy and as tools to study osteoclast biology. In our rational design, two focused libraries were constructed, in each of which an Arginine-Glycine-Aspartic acid (RGD) sequence (a motif crucial for α_v_β_3_ integrin ligand binding) flanked by random amino acids was introduced into the dimerization interface of the M-CSF ligand with the aim to change its activity from agonistic to antagonistic. This modification blocks the interactions between two M-CSF monomers and hence their subsequent homodimerization and activation of the M-CSF/c-FMS signal transduction cascade (Fig 1). Our combinatorial strategy to further develop this unique antagonist involved the screening of the abovementioned focused libraries against α_v_β_3_ integrin. Thereafter, functional FACS-based screening assays were applied to quantitatively select for M-CSF mutants with the highest expression, stabilities and affinities for both α_v_β_3_ integrin and c-FMS. With this approach, we generated a number of unique dual-specific M-CSF mutants capable of binding to and antagonizing the biological activity of both c-FMS and α_v_β_3_ integrin *in vitro* and *in vivo*.

## Results

### Testing the compatibility of the M-CSF/c-FMS system with the yeast surface display method

The M-CSF glycoprotein is secreted as a homodimer that, upon binding to two c-FMS receptors, induces c-FMS autophosphorylation followed by activation of downstream signaling pathways [23]. Our strategy for converting M-CSF from an agonist into an antagonist of the c-FMS receptor relied on impairing the ability of M-CSF to dimerize. To this end, we exploited the critical role of the cysteine in position 31 in the covalent dimerization of wild-type M-CSF (M-CSF_WT_) [24], namely, we generated a mutant M-CSF with impaired covalent dimerization capability (designated M-CSF_C31S_) by replacing C31 with a serine residue (S1A Fig). To determine whether the M-CSF_C31S_/c-FMS system was indeed compatible with the yeast surface display (YSD) method, we transformed the M-CSF_C31S_ gene into *Saccharomyces cerevisiae* and determined M-CSF_C31S_ protein expression (S2A and S2B Fig) and binding to soluble c-FMS (S2C Fig). To evaluate the binding affinity of M-CSF_C31S_ for c-FMS in the YSD system, we measured its apparent binding to different concentrations of soluble c-FMS (k_D,app_ = 20 nM; S2D Fig). The observed K_D,app_ did indeed resemble the K_D_ of 13.6 nM known from the literature for the M-CSF homodimer [25], thereby validating the compatibility of YSD with the M-CSF_C31S_/c-FMS system.

### Engineering and selection of M-CSF harboring RGD motif proteins with high affinity for c-FMS and α_v_β_3_ integrin

**Fig 2:**
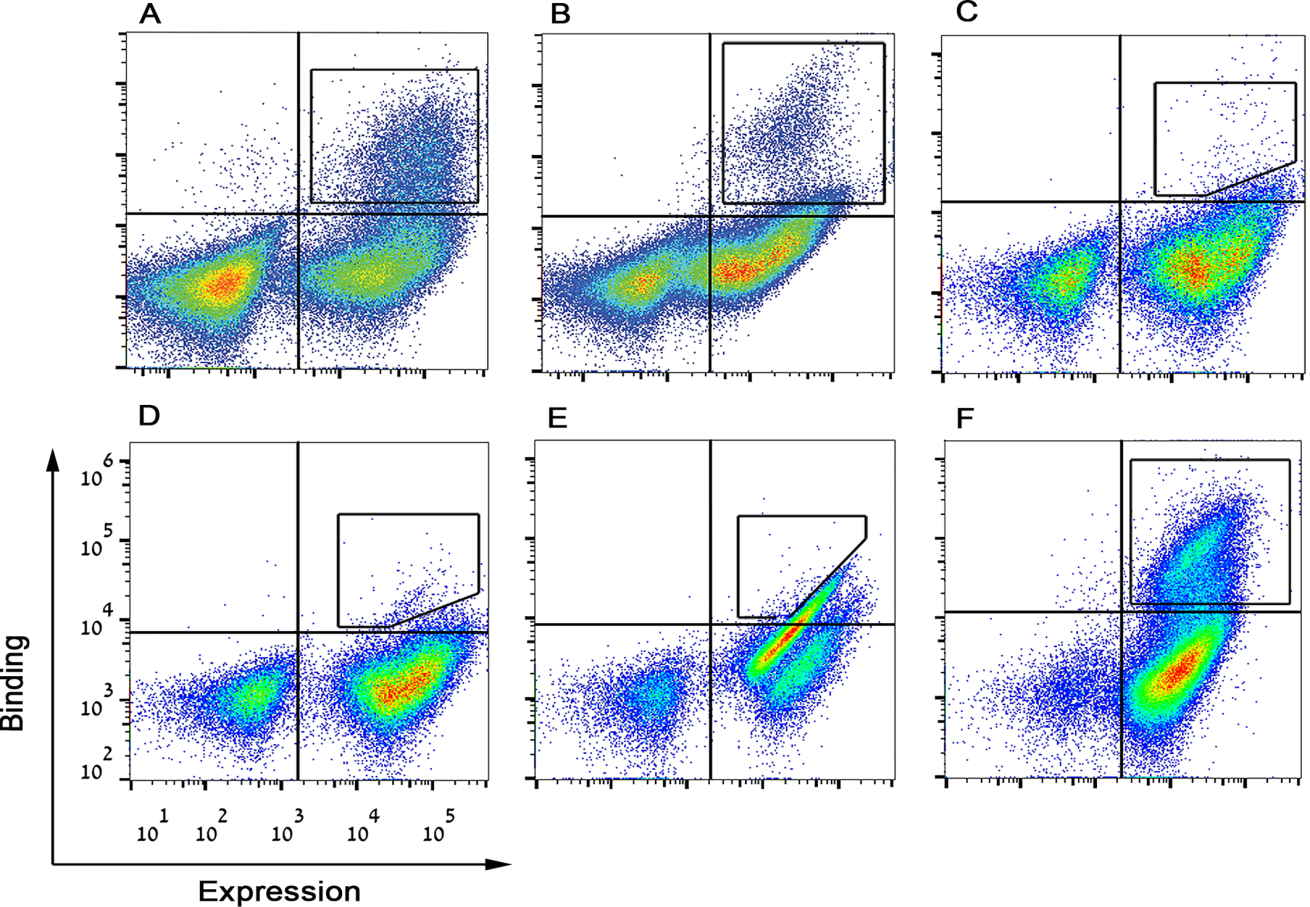
FACS dot plot of M-CSF_RGD_ affinity maturation process. Yeast-displayed mutant pools were tested for binding to (A) 200 nM c-FMS, (B) 500 nM α_v_β_3_ integrin, (C) 250 nM α_v_β_3_ integrin, (D) 100 nM α_v_β_3_ integrin, (E) 20 nM α_v_β_3_ integrin, and (F) 50 nM c-FMS. High target binders were sorted as indicated in each figure with black square- or polygon-shaped gates. M-CSF, macrophage colony-stimulating factor; RGD, Arginine-Glycine-Aspartic acid.

To construct a dual-specific M-CSF monomer that can bind both c-FMS and α_v_β_3_ integrin, two libraries were created by replacing one of two loops on the M-CSF_C31S_ scaffold with RGD, flanked by three random amino acids on each side, namely, by XXXRGDXXX, where X represents any random amino acid (designated M-CSF_RGD_) (S1B and S1C Fig). To identify and isolate M-CSF variants with high affinity for both c-FMS and α_v_β_3_ integrin, we generated a YSD construct (S3 Fig) that allowed us to separate high-affinity clones from low-affinity clones by using flow cytometry. After confirming that M-CSF_RGD_ libraries were well expressed on the surface of the yeast cell wall and that these libraries did indeed bind 100 nM soluble c-FMS and 500 nM α_v_β_3_ integrin (S4 Fig), we merged the two libraries and regarded them as a single library that consisted of 1.1 × 10^7^ variants. For affinity maturation, the M-CSF_RGD_ library was sorted in a stepwise manner against α_v_β_3_ integrin and c-FMS by using FACS (Fig 2 and S5 Fig). To identify the specific variants that would bind both c-FMS and α_v_β_3_ integrin, we tested individual clones for c-FMS and α_v_β_3_ integrin binding and identified three unique clones with high affinity for both receptors (S6 Fig). These M-CSF_RGD_ variants were designated 4.22, 4.24 (both from sort four), and 5.6 (from sort five) (S1F Fig). Selective binding of these three variants to α_v_β_3_ integrin and not to other integrins was confirmed by evaluating their binding to α_v_β_3_, α_4_β_7_, α_iib_β_3_ and α_5_β_1_ integrins (250 nM) (S6 Fig). Since all three variants showed high specificity for α_v_β_3_ integrin, they were regarded as good candidates for osteoclast-specific drug development, since osteoclasts express high levels of α_v_β_3_ integrin as early as 48 h of differentiation [26].

### Production, purification, and characterization of bispecific M-CSF_RGD_ variants and monospecific M-CSF controls

We produced the bispecific M-CSF_RGD_ protein variants and their monospecific controls, M-CSF_c-FMS_ (S1E Fig) and M-CSF_αvβ3_ (S1D Fig), with the aim to characterize these proteins in their soluble forms. The proteins were expressed in *Pichia pastoris*, glycans were removed, and the nonglycosylated proteins were purified by using affinity and size exclusion chromatography, giving the expected size of approximately 21 kDa (S7A and S7E Fig). Circular dichroism (CD) spectra of the purified proteins showed similar curves, corresponding to a protein that consists mostly of α-helix motifs, which means that the RGD loops did not cause a significant change in the protein secondary structure (S7C Fig). The CD spectra were also exploited to determine the melting temperatures of the proteins, giving 73 °C for variant 4.22, 77.5 °C for variant 4.24, 85 °C for variant 5.6, and 68.7 °C for M-CSF_c-FMS_; the melting temperature for M-CSF_αvβ3_ could not be determined (S7D Fig). The exact molecular weights of the purified variants and monospecific controls, determined by mass spectrometry, were as follows: 21.7 kDa for both variant 4.22 (S7B Fig) and variant 4.24, 22 kDa for variant 5.6, 21.8 kDa for M-CSF_c-FMS_, and 21.7 kDa for M-CSF_αvβ3_.

To ensure that our purified proteins did not dimerize and would thus be able to inhibit c-FMS dimerization and downstream signaling, we incubated the proteins with the cross-linker reagent BS^3^ (bis[sulfosuccinimidyl]suberate) to enhance noncovalent intermolecular interactions and to enable visualization of these interactions. As expected, the three bispecific M-CSF_RGD_ proteins as well as the monospecific M-CSF_αvβ3_ and M-CSF_c-FMS_ proteins did not dimerize even at a very high BS^3^ concentration of 2500 μM (S8 Fig). In contrast, M-CSF_WT_ did dimerize at the very low BS^3^ concentration of 25 μM. Since M-CSF_RGD_ and M-CSF_c-FMS_ do not dimerize in solution, they are expected to bind c-FMS and prevent its dimerization and activation.

### Purified bispecific proteins bind c-FMS and α_v_β_3_ integrin with different affinities but do not bind other RGD binding integrins

**Fig 3:**
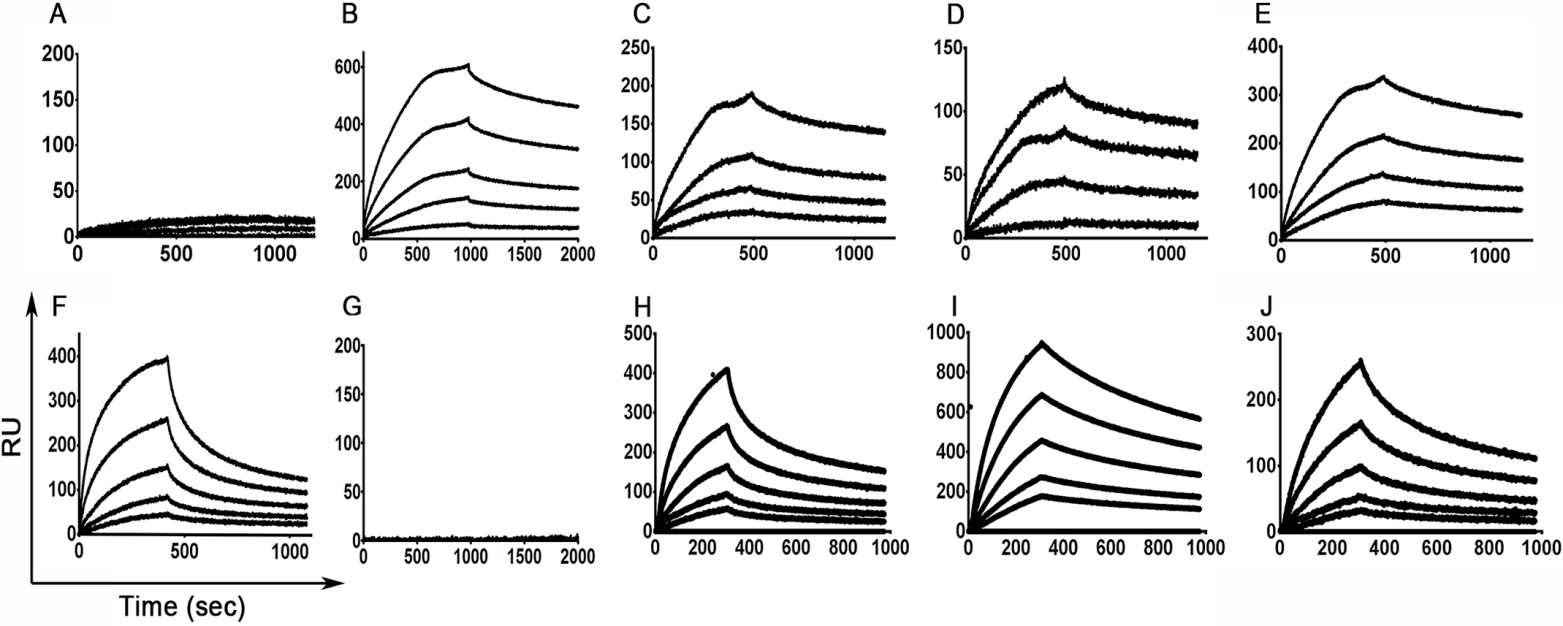
SPR binding sensorgrams of the different purified M-CSF proteins. Binding of M-CSF_αvβ3_ (A and F), M-CSF_c-FMS_ (B and G), 4.22 (C and H), 4.24 (D and I), and 5.6 (E and J) to c-FMS (A-E) and α_v_β_3_ integrin (F-J) at concentrations of 12.5 nM, 25 nM, 50 nM, 100 nM, and 200 nM. Source data can be found in S1 Data. M-CSF, macrophage colony-stimulating factor; RUs, response units; SPR, surface plasmon resonance.

An optimal competitor for native protein receptors should exhibit a higher binding affinity (lower K_D_) than the binding affinities of their natural ligands. The K_D_ of M-CSF_WT_ to its c-FMS receptor lies in the low nanomolar range (13.6 nM) [25]. The binding affinity of α_v_β_3_ integrin to its natural ligand, vitronectin, is 40.7 nM [27]. The binding affinities of M-CSF_RGD_, M-CSF_αvβ3_, and M-CSF_c-FMS_ to c-FMS and α_v_β_3_ integrin were obtained from the equilibrium-binding phase of the sensorgrams measured by surface plasmon resonance (SPR) (S9 Fig). The affinities of the protein variants to c-FMS were lower than those of M-CSF_WT_ as a result of the RGD loop substitution, resulting in K_D_ values of 152 nM for 4.22, 219 nM for 4.24, 96 nM for 5.6, and 195 nM for M-CSF_c-FMS_ (Fig 3 and S1 Table). As expected, M-CSF_αvβ3_ did not bind to c-FMS. The binding affinities of the M-CSF_RGD_ proteins to α_v_β_3_ integrin were 199 nM for 4.22, 108 nM for 4.24, and 245 nM for 5.6, and the K_D_ of M-CSF_αvβ3_ was 231 nM. As expected, M-CSF_c-FMS_ did not bind to α_v_β_3_ integrin. Thus, consistent with the YSD results, the three M-CSF_RGD_ proteins demonstrated different affinities for α_v_β_3_ integrin and c-FMS: 4.24 had a low affinity for α_v_β_3_ integrin and a higher affinity for c-FMS, 5.6 exhibited high affinity for c-FMS and lower affinity for α_v_β_3_ integrin, and 4.22 had intermediate affinity levels for the two receptors. This heterogeneity in binding affinities underlines the plasticity of ligand-based bispecific antagonists, which can provide the most sensitive balance of efficacy and specificity. This binding heterogeneity may also enable us to predict the contribution of each of the two targets, c-FMS and α_v_β_3_ integrin, to osteoclast activation and differentiation (see following sections).

To determine the specificity of the three M-CSF_RGD_ variants for α_v_β_3_ integrin vis-à-vis the other integrins produced in the human body, SPR was used to evaluate the binding of M-CSF_RGD_ variants (1 μM) to four different RGD-binding integrins (α_3_β_1_, α_4_β_7_, α_5_β_1_, and α_v_β_3_). Consistent with our previous results, α_v_β_3_ integrin bound the soluble M-CSF_RGD_ proteins with high affinity (S10A Fig). Integrins α_3_β_1_, α_4_β_7_, and α_5_β_1_ did not bind to the three M-CSF_RGD_ proteins (S10B–S10D Fig).

### Computational docking model of α_v_β_3_ integrin and M-CSF_RGD_/c-FMS complex predicts the interaction interface

**Fig 4:**
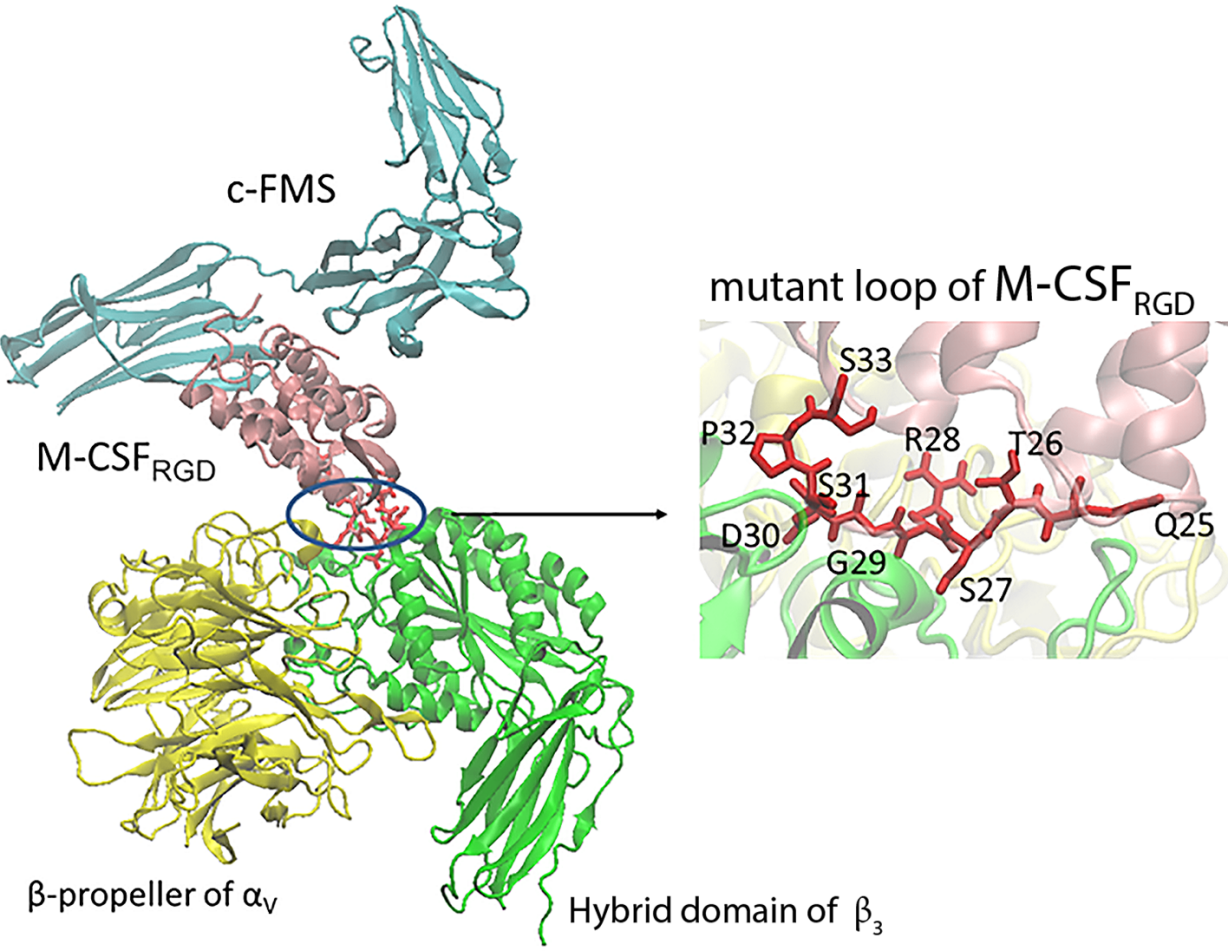
Docking model of the M-CSF_RGD_ variant 4.22/c-FMS-α_v_β_3_ integrin complex. M-CSF_RGD_ variant 4.22 is shown in pink, c-FMS in cyan, α_v_ in yellow, β_3_ in green, and QTSRGDSPS mutant residues in red. M-CSF, macrophage colony-stimulating factor; RGD, Arginine-Glycine-Aspartic acid.

To better understand the interactions between M-CSF_RGD_ variant 4.22 and α_v_β_3_ integrin, a molecular docking procedure was performed with PatchDock [28] both for the M-CSF_C31S_ (S11 Fig) and 4.22 with the binding domains of α_v_β_3_ integrin. For further refinement, clustering was performed on the docking solutions, and the most abundant structure was used for the analysis of the model. The dimerization loop 1 of M-CSF is located on the opposite side of the molecule to the c-FMS binding domain. Thus, monomeric M-CSF_RGD_ is capable of simultaneous binding to both c-FMS and α_v_β_3_ integrin (Fig 4), since there is no steric clash between the native receptor c-FMS and α_v_β_3_ integrin. In spatial terms, the ligand could thus serve as a bispecific agent for both receptors.

R28 of RGD is the only residue contacting the α_v_ subunit. The rest of the loop is located closer to β_3_. Almost all the mutant residues lie on the β_3_ surface, making contact with integrin residues but still exposed to the surroundings (S12 Fig). This configuration creates a total interaction surface of 1,483 Å^2^ between M-CSF_RGD_ variant 4.22 and α_v_β_3_ integrin. The differences in RGD orientation in comparison to the crystal structure of α_v_β_3_ integrin in a complex with cyclic RGD (cRGD) [29] are shown in S13 Fig. The D side of the ligand is buried deeper in the β_3_ subunit in our model than in the crystal. The location of G in the middle is similar in both structures, while R points in a different direction and thus has a very limited contact with α_v_.

### M-CSF_RGD_ variants bind to both c-FMS and α_v_β_3_ integrin expressed on cells

**Fig 5:**
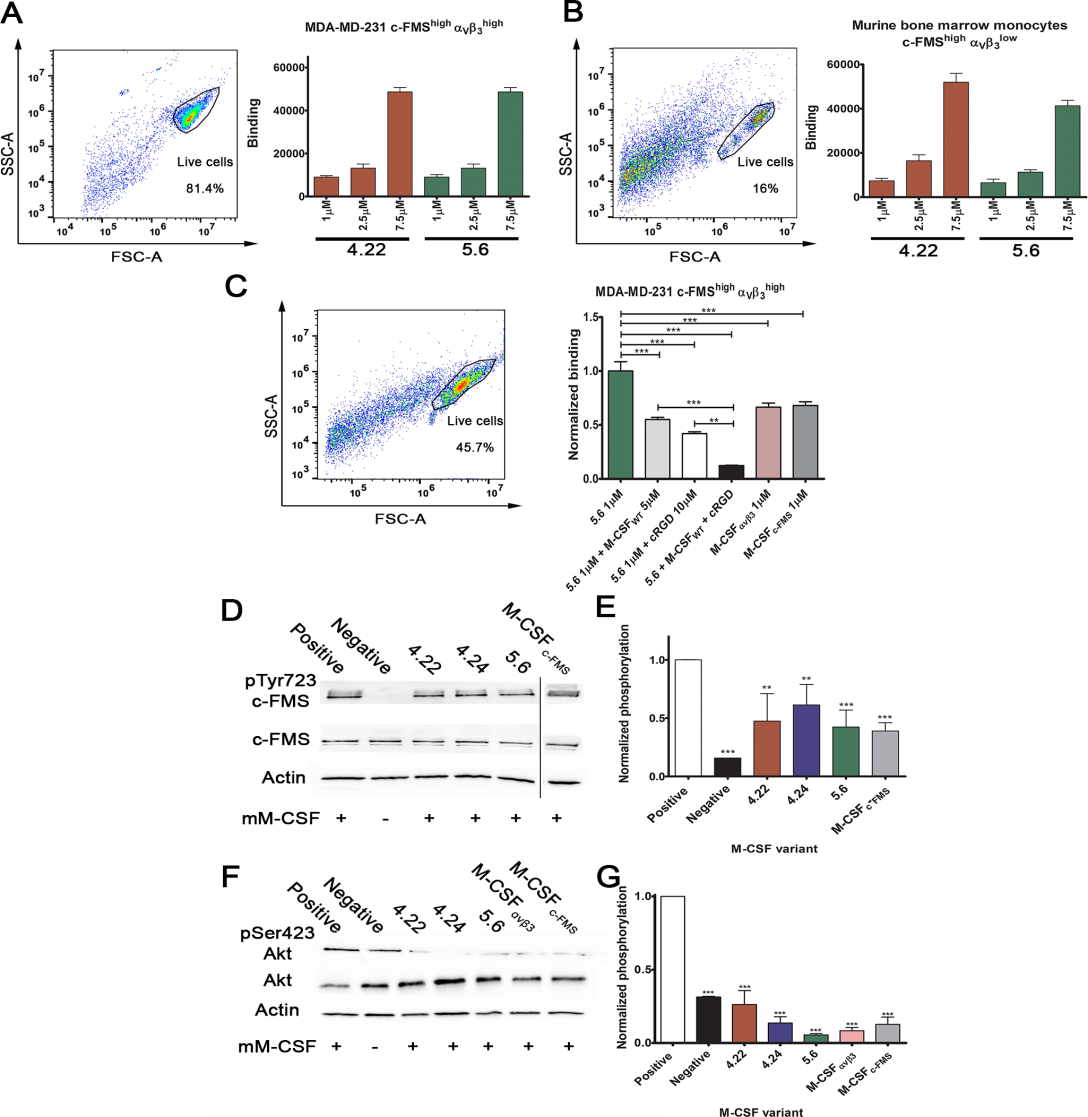
Cell binding, c-FMS, and Akt activation assays. Purified M-CSF_RGD_ variants 4.22 and 5.6 were tested for binding to (A) MDA-MB-231 breast cancer cell line and (B) murine BMMs at different protein concentrations (1 μM, 2.5 μM, and 7.5 μM). The cellular expression levels of c-FMS and α_v_β_3_ integrin are indicated as superscripts. (C) Cell competition binding assay for variant 5.6 in the presence and absence of the two competitors, namely 10 μM cRGD and 5 μM M-CSF_WT_. Binding of M-CSF_c-FMS_ and M-CSF_αvβ3_ to the cells is shown for comparison. (D) Tyrosine phosphorylation of c-FMS and (F) serine phosphorylation of Akt in murine BMMs. Different gel runs are separated by a black line. (E) Relative c-FMS and (G) Akt phosphorylation levels of BMMs following incubation with M-CSF_c-FMS_, M-CSF_αvβ3_, and M-CSF_RGD_ variants 4.22, 4.24, and 5.6 in the presence of recombinant M-CSF as a competitor. Chemiluminescence read-outs were quantified by densitometry. Data are means ± SEM of triplicates. *p < 0.05, **p < 0.01, ***p < 0.001. The aspect ratios of the membranes in panels D and F were changed. Source data and analysis can be found in S2 Data. BMM, bone-marrow–derived monocyte; cRGD, cyclic RGD; M-CSF, macrophage colony-stimulating factor; MDA-MB-231, MD Anderson metastatic breast 231; RGD, Arginine-Glycine-Aspartic acid.

Assays for direct cell binding were performed on breast cancer cell line MD Anderson metastatic breast 231 (MDA-MB-231) and murine bone-marrow–derived monocytes (BMMs) for the following reasons. MDA-MB-231 cells express human α_v_β_3_ integrin and c-FMS on the cell membrane in a protein expression pattern that imitates the osteoclast state just before reaching full differentiation and maturation (S14C and S14D Fig). This cell line showed binding to 1 μM, 2.5 μM, and 7.5 μM of each of the M-CSF_RGD_ variants 4.22 and 5.6 in a dose-dependent manner (Fig 5A), with variant 5.6 showing similar binding to 4.22. Murine BMMs express high levels of c-FMS and low levels of α_v_β_3_ integrin (S14A and S14B Fig), a state that imitates an early osteoclast differentiation stage. For these cells, variants 4.22 and 5.6 showed similar dose-dependent binding. To show that M-CSF_RGD_ variants do indeed bind to c-FMS and α_v_β_3_ integrin expressed on cells, we used specific competitors to these receptors, namely, cRGD, which will compete with α_v_β_3_ integrin binding, and M-CSF_WT_, which will compete with c-FMS binding. M-CSF_RGD_ variant 5.6 showed reduced binding to MDA-MB-231 in the presence of each competitor versus in the absence of inhibitors. Strikingly, the binding to the cells was significantly reduced in the presence of both inhibitors, thereby emphasizing that the ability to bind both receptors contributes to the total binding capability of the bispecific proteins. Moreover, the fact that the binding of variant 5.6 to the cells was stronger than that of M-CSF_c-FMS_ and M-CSF_αvβ3_ confirmed the advantage of the bispecific protein over the monospecific proteins (Fig 5C).

### M-CSF_RGD_ variants inhibit induction of Akt and c-FMS phosphorylation

Upon binding to c-FMS, M-CSF_WT_ induces c-FMS phosphorylation, which is essential for downstream signaling. To test whether binding of M-CSF_RGD_ inhibits the activation of c-FMS, we monitored its phosphorylation in the presence and absence of recombinant M-CSF in murine BMMs. As expected, the variants did not activate c-FMS (S15A Fig), and the inhibition of c-FMS phosphorylation correlated with the affinity of the different M-CSF_RGD_ proteins for c-FMS, with variant 5.6 (which binds c-FMS with the highest affinity) being the most efficient inhibitor and variant 4.24 (which has the lowest c-FMS binding affinity) the least efficient (Fig 5D and 5E). These data show the correlation between inhibition of c-FMS by the M-CSF_RGD_ proteins and the binding affinity of the variant proteins to c-FMS. It is well known that human M-CSF_WT_ can bind mouse c-FMS, but the binding affinity is much lower than that for mouse M-CSF to mouse c-FMS [30]. Nonetheless, the M-CSF_RGD_ variants significantly inhibited c-FMS phosphorylation and successfully competed with murine M-CSF in cells expressing murine c-FMS even though the M-CSF_RGD_ variants were of human origin. This finding suggests that the M-CSF_RGD_ variants should be highly effective as antagonists even in mouse cell models. The same experiment was performed for Akt activation with and without recombinant M-CSF. Akt activation has previously been shown to prolong cell survival in response to c-FMS and α_v_β_3_ integrin activation [31]. As expected, our protein variants did not spontaneously activate Akt phosphorylation (S15B Fig), and they significantly inhibited M-CSF–induced Akt phosphorylation (Fig 5F). The M-CSF_RGD_ variants, M-CSF_c-FMS_, and M-CSF_αvβ3_ inhibited Akt phosphorylation, with the normalized phosphorylation levels being lower than the basal level of the negative control (Fig 5G).

### M-CSF_RGD_ inhibits actin belt formation in mature osteoclasts

**Fig 6:**
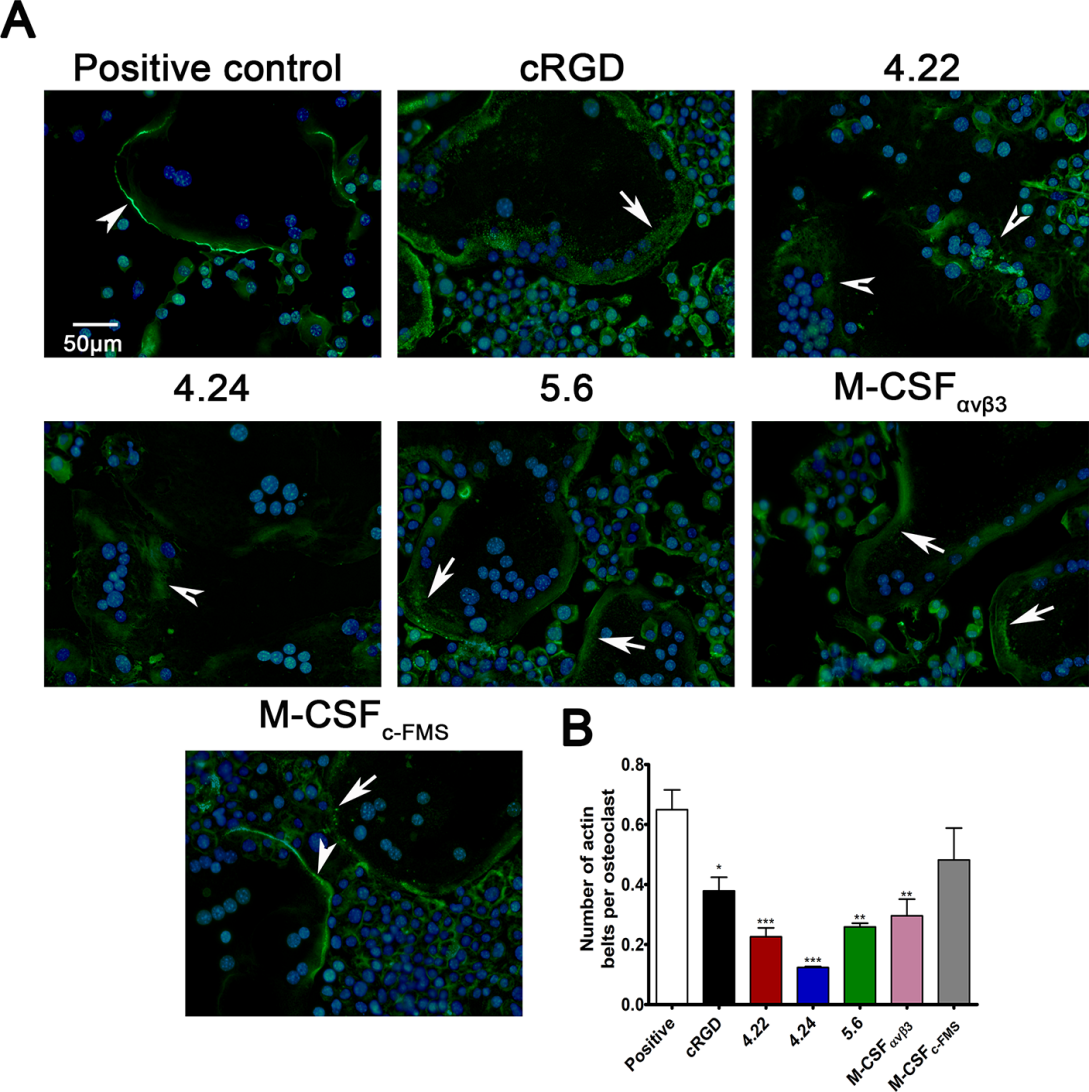
Actin belt formation in mature osteoclasts incubated with M-CSF_RGD_ variants. (A) Murine BMMs were allowed to differentiate into osteoclasts in the presence of M-CSF and RANKL for 72 h. Then, the cells were incubated for an additional 24 h without (positive control) or with inhibitors (5 μM), followed by fixation and staining for F-actin and nuclei. The cells formed solid actin belts (white arrowheads), actin belts with "scattered" podosomes (white arrows), or "amorphous" actin stain distribution (barbed arrowheads). (B) The actin belts’ formation was quantified by normalizing the numbers of solid actin belt to the number of osteoclasts. Pictures are representatives of 35 images acquired from five different wells per sample. Data are means ± SEM of triplicates. *p < 0.05, **p < 0.01, ***p < 0.001. Source data can be found in S3 Data. BMM, bone-marrow–derived monocyte; cRGD, cyclic RGD; M-CSF, macrophage colony-stimulating factor; RANKL, receptor activator of the nuclear factor–kappa-B ligand; RGD, Arginine-Glycine-Aspartic Acid.

For a mature osteoclast to resorb bone properly, it must form a unique structure termed the "sealing zone"; this structure consists of an actin ring made from a dense actin mesh connected by adhesion complexes called podosomes. The formation of the actin ring involves several stages: at the beginning, podosomes are formed throughout the cell in a scattered manner; then groups of podosomes form small actin structures, known as belts, and upon osteoclast maturation, the belts move to the cell periphery, cluster, and form an actin ring. Podosomes contain high levels of α_v_β_3_ integrin, which is essential for the formation on bones of proper actin rings with condensed podosome networks [32, 33]. As opposed to cells grown on bone, osteoclasts cultured on glass produce actin belts but are unable to form stable actin rings. However, the preceding stages of the podosome network are similar for cells grown on bone and on glass and are known to be regulated by α_v_β_3_ integrin [34]. To test whether the inhibition of α_v_β_3_ integrin by bispecific M-CSF_RGD_ variants affects the organization of podosome networks, mouse BMMs cultured on glass were allowed to differentiate into mature osteoclasts to the point that multinucleated cells were formed and exhibited an actin belt structure. At this point, the inhibitors were added, and the cells were incubated for an additional 24 h before they were fixed and stained. Cells incubated only with M-CSF and RANKL without any inhibitor (i.e., positive control) formed a dense continuous actin belt structure. Addition of the monospecific M-CSF_c-FMS_ did not drastically inhibit actin belt formation and abundance, and osteoclasts presenting dense continuous actin belts were observed (Fig 6A and 6B); nevertheless, scattered podosomes presenting immature belts were more abundant than in the positive controls (Fig 6, lower panel). Addition of either cRGD peptide, a well-established α_v_β_3_ integrin inhibitor [35], or the monospecific M-CSF_αvβ3_ decreased actin belts’ distribution (Fig 6B). The actin belts in cells incubated with these inhibitors had wider and fainter actin stain, probably as a failure of the podosomes to form condensed structures as higher distribution of scattered podosomes was observed in these cells. The bispecific M-CSF_RGD_ variant 5.6, which exhibited the lowest affinity to α_v_β_3_ integrin, showed the same phenotype as cRGD and M-CSF_αvβ3_. Strikingly, the bispecific M-CSF_RGD_ variants 4.22 and 4.24 had the highest impact on actin belt formation and density. Dense actin structures and even scattered podosomes were hard to detect, and instead, the actin stain in osteoclasts cultured in the presence of these inhibitors had a diffusible appearance throughout the cell (Fig 6, S16 Fig).

### M-CSF_RGD_ variants inhibit osteoclast differentiation *in vitro*

**Fig 7:**
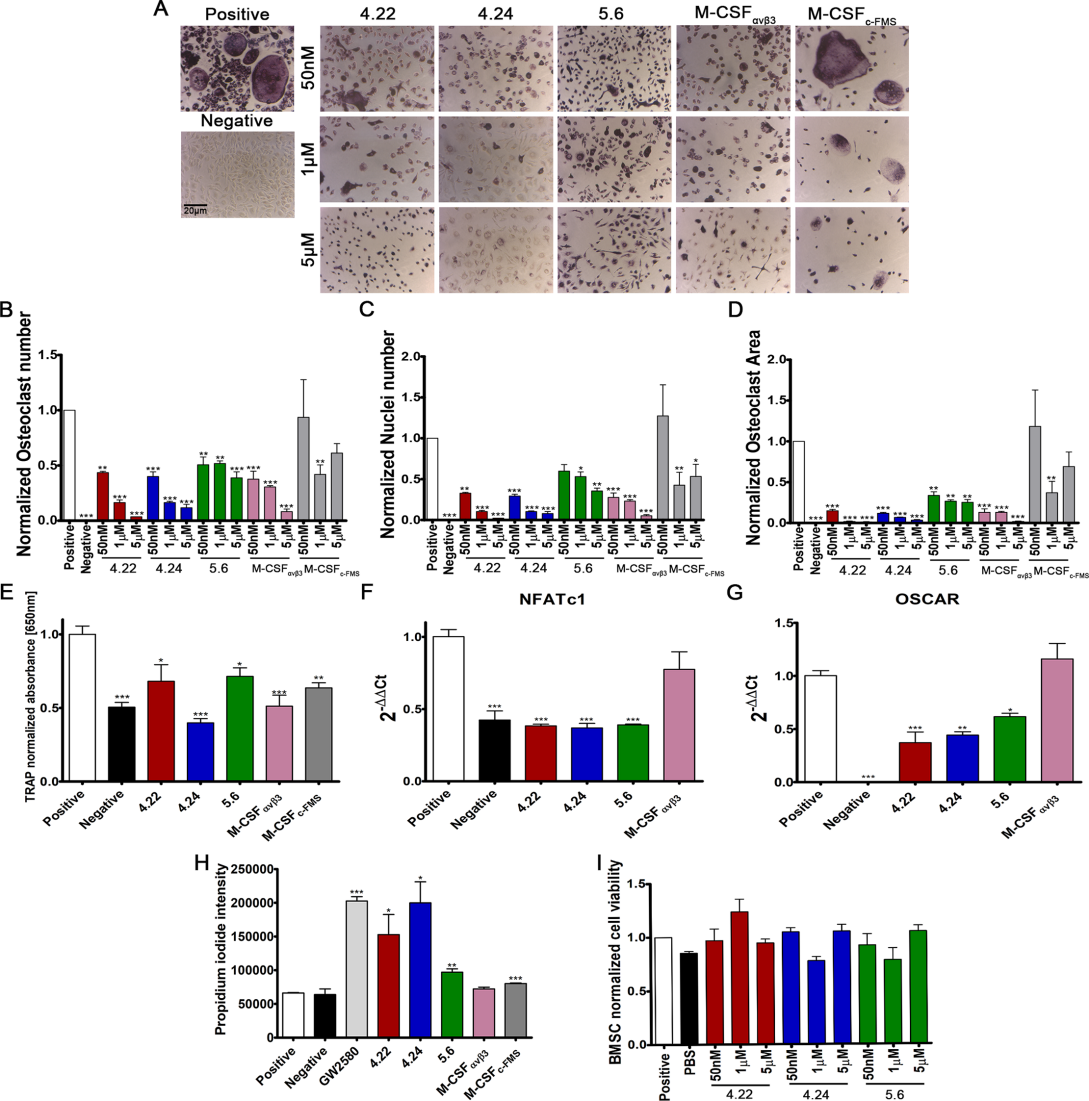
Effects of M-CSF_RGD_ variants on murine osteoclast differentiation. Murine BMMs were cultured for 96 h in a medium containing recombinant mouse M-CSF (20 ng/ml), RANKL (20 ng/ml), and different concentrations of inhibitors. The same medium without inhibitors was used as a positive control. The medium for the negative control was supplemented with recombinant M-CSF. (A) Cells were fixed and stained for TRAP. (B–D) Cells were examined for: (B) number of osteoclasts, (C) number of nuclei within osteoclasts, (D) total surface area, and (E) total TRAP absorbance were normalized to the positive control. The effect of the inhibitors on markers of osteoclast differentiation was assessed using quantitative PCR for (F) NFATc1 and (G) Oscar mRNA expression. (H) The effect of the inhibitor on pre-osteoclast cell survival was assayed by measuring PI incorporation in osteoclasts cultured for 48 h in the presence of 1 μM of each inhibitor. (I) To test whether there is an unspecific toxic effect of the inhibitors, BMSCs were tested for cell viability in the XTT assay in the presence of three different concentrations of inhibitor (50 nM, 1 μM, and 5 μM). Data are means ± SEM of triplicates. A total of 2,340 frames were analyzed for 1,581 osteoclasts and 5,313 nuclei. *p < 0.05, **p < 0.01, ***p < 0.001. Source data and analysis can be found in S4 Data. BMM, bone-marrow–derived monocyte; BMSC, bone-marrow–derived mesenchymal stromal cell; M-CSF, macrophage colony-stimulating factor; PI, propidium iodide; RANKL, receptor activator of the nuclear factor–kappa-B ligand; RGD, Arginine-Glycine-Aspartic acid; TRAP, tartrate-resistant acid phosphatase; XTT, 2,3-bis-(2-methoxy-4-nitro-5-sulfophenyl)-2H-tetrazolium-5-carboxanilide.

**Fig 8:**
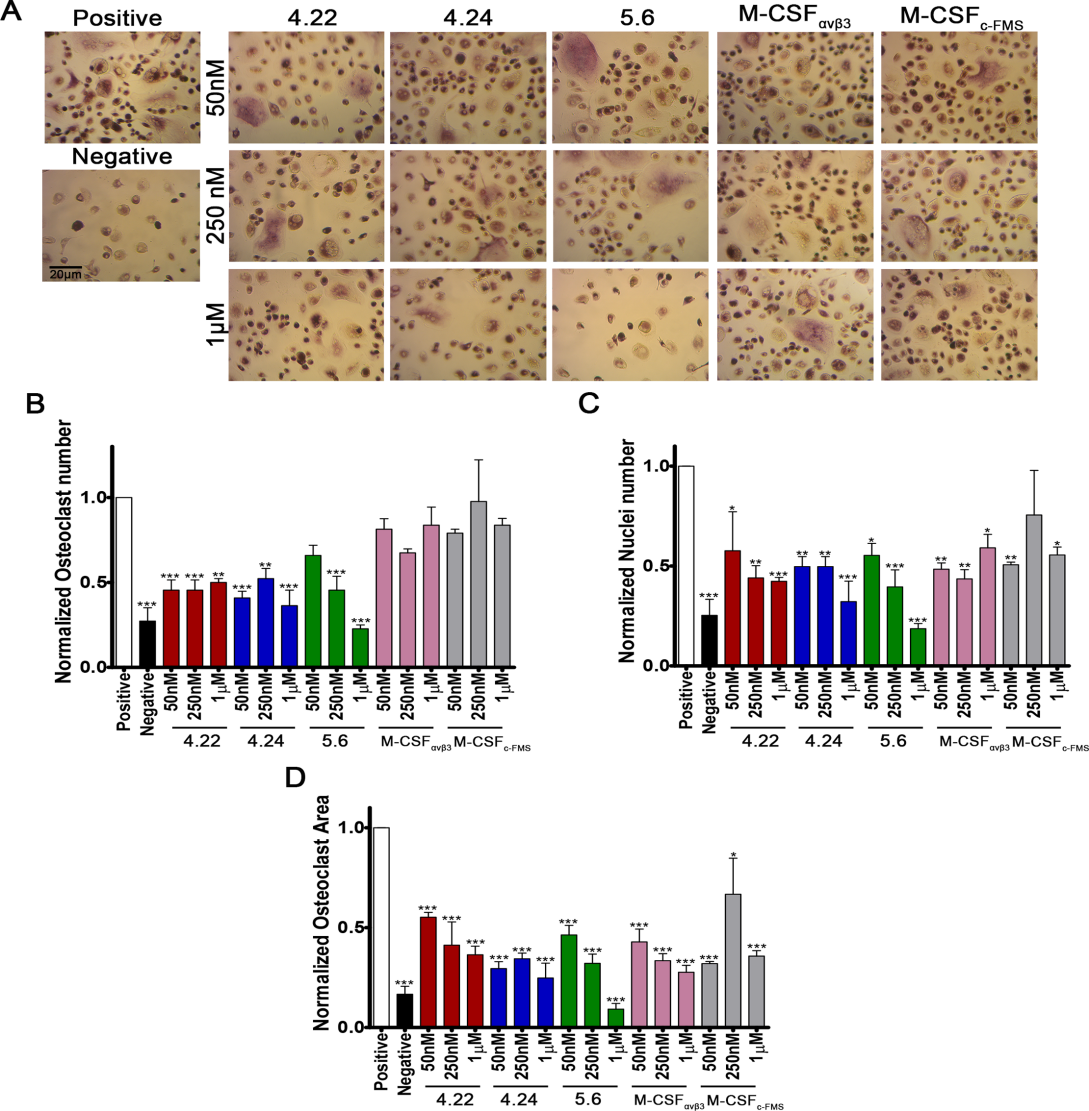
Effects of M-CSF_RGD_ variants on human osteoclast differentiation. Human CD14^+^ cells were cultured for 96 h in medium containing recombinant human M-CSF (20 ng/ml), murine RANKL (20 ng/ml), and different concentrations of inhibitors. The same medium without inhibitors was used as a positive control. The αMEM medium for the negative control was supplemented with recombinant M-CSF. (A) Cells were fixed and stained for TRAP. (B-D) Cells were examined for (B) number of mature osteoclasts, (C) number of nuclei within osteoclasts, and (D) total surface area. Results were normalized to the positive control. Data are means ± SEM of triplicates. A total of 1,620 frames were analyzed for 477 osteoclasts and 1,897 nuclei. *p < 0.05, **p < 0.01, ***p < 0.001. Source data and analysis can be found in S5 Data. αMEM, alpha Minimum Essential Medium; M-CSF, macrophage colony-stimulating factor; RANKL, receptor activator of the nuclear factor–kappa-B ligand; RGD, Arginine-Glycine-Aspartic acid; TRAP, tartrate-resistant acid phosphatase.

To assess the ability of the three bispecific M-CSF_RGD_ variants and their monospecific controls, M-CSF_c-FMS_ and M-CSF_αvβ3_, to inhibit osteoclast differentiation, we monitored the effects of elevated concentrations of these proteins in cultures of differentiating BMMs or human peripheral blood CD14^+^ monocytes. The proteins were added together with recombinant RANKL and human (for CD14^+^ cells) or murine (for BMMs) M-CSF to initiate osteoclast differentiation. We note that as osteoclasts differentiate, the number of nuclei and the surface area increase as a result of cell fusion and cytoskeletal rearrangements, respectively. Strikingly, in cultures of differentiating BMMs and CD14^+^ cells, all three M-CSF_RGD_ variants inhibited the nuclei number, surface area, total osteoclast number, and tartrate-resistant acid phosphatase (TRAP) absorbance, with inhibition being significant at a concentration as low as 50 nM (Fig 7 and Fig 8). To further validate the ability of the M-CSF_RGD_ variants to inhibit osteoclast differentiation, the mRNA expression levels of osteoclast-associated receptor (OSCAR) and NFATc1—two well-known osteoclast differentiation markers exhibiting increased expression during osteoclast differentiation—were evaluated [36, 37]. The three M-CSF_RGD_ variants significantly inhibited OSCAR and NFATc1 expression while M-CSF_αvβ3_ had no inhibitory effect (Fig 7F and 7G). In general, the ability of the M-CSF monospecific controls to inhibit osteoclast differentiation was inferior to that of the bispecific M-CSF_RGD_ variants, particularly in the human CD14^+^ cell cultures. Since M-CSF signaling is known to regulate osteoclast survival, inhibition of M-CSF is expected to lead to a decrease in osteoclast survival. Indeed, a smaller number of cells was observed in osteoclasts cultured in the presence of the M-CSF_RGD_ variants. In addition, an assay for cell death, based on propidium iodide (PI) permeability, in differentiating osteoclast cultures showed that the three M-CSF_RGD_ inhibitors and the M-CSF_c-FMS_ significantly increased cell death versus the small molecule GW2580 (a known inhibitor for c-FMS phosphorylation) used in the control assay. We note that M-CSF_αvβ3_ was unable to induce cell death, probably because of its inability to bind c-FMS (Fig 7H). To rule out nonspecific toxic effects of the M-CSF_RGD_ variants on cell proliferation and survival, we added these inhibitors to murine bone-marrow–derived mesenchymal stromal cells (BMSCs), whose physiological microenvironment is the same as that of BMMs. No changes in survival and proliferation capabilities of BMSCs cultured with elevated concentrations of the M-CSF_RGD_ variants were observed (Fig 7I). Taken together, these results show that all M-CSF_RGD_ variants are highly efficient in inhibiting osteoclast differentiation and spreading even at a low concentration and have no apparent toxic effects on other primary bone-marrow–residing cells.

### *In vivo* biodistribution of M-CSF_RGD_ variant 5.6

**Fig 9:**
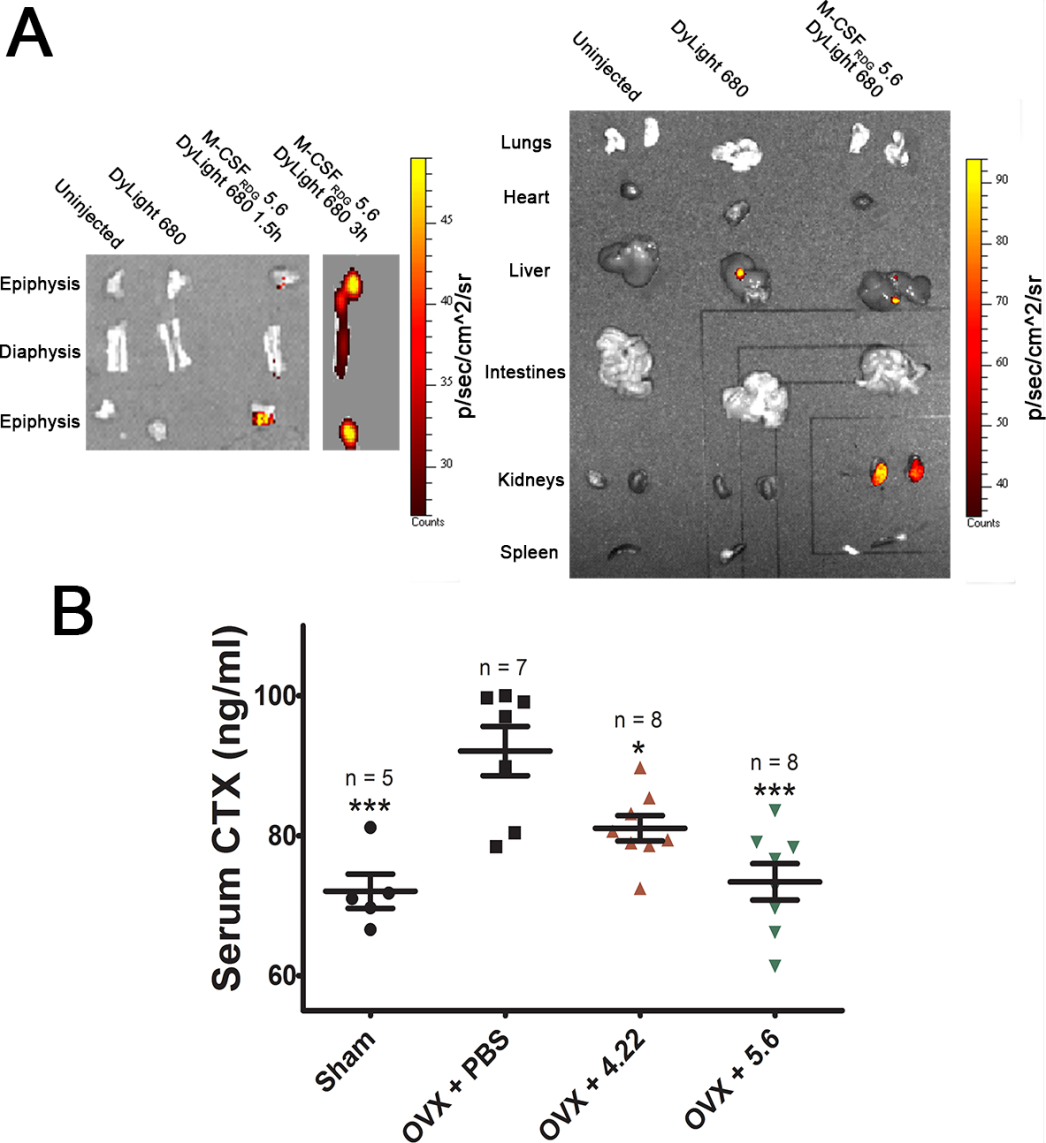
M-CSF_RGD_ proteins inhibit bone resorption in ovariectomized mice. (A) *Ex vivo* images of 12-weeks-old WT C57BL6 mice organs. The organs were removed and imaged 1.5 h and 3 h after mice were injected s.c. and compared with organs of a mouse that had been injected with unconjugated dye and of a mouse that had not been injected with the proteins (right). To determine whether M-CSF_RGD_ variant 5.6 accumulates in the bones, the epiphysis and diaphysis of the femur of a mouse injected with variant 5.6 were compared with those of the control mice (left). (B) Ten-weeks-old mice were ovariectomized, and starting 2 wk after the surgery, they were injected twice a day with PBS or M-CSF_RGD_ variants 4.22 or 5.6 for 3 d. Thereafter, serum CTX-I levels were determined by ELISA. Data are means ± SEM. *p < 0.05, **p < 0.01, ***p < 0.001. Source data and its analysis can be found in S6 Data. CTX-I, carboxy-terminal telopeptide of type I collagen; ELISA, enzyme linked immunosorbent assay; M-CSF, macrophage colony-stimulating factor; OVX, ovariectomy-induced bone loss; PBS, phosphate-buffered saline; RGD, Arginine-Glycine-Aspartic acid; s.c., subcutaneously; WT, wild type.

To evaluate the biodistribution of M-CSF_RGD_, we chose variant 5.6 because yields of this purified protein were higher than those for purified variants 4.22 and 4.24. The protein was stained with DyLight 680-NHS (N-hydroxysuccinimide) Ester, and 2 nmol of stained variant 5.6 or unconjugated dye were injected subcutaneously (s.c.) into wild-type (WT) C57BL6 mice. The mice were killed 1.5 h and 3 h after injection, their organs were removed, and the accumulation of variant 5.6 in the organs was compared to that in a control untreated mouse. Variant 5.6 accumulated in the kidneys and the bladder as part of the natural clearance and not in other organs, such as the heart, liver, and spleen (Fig 9A, right). Importantly, examination of the epiphysis and diaphysis of a C57BL6 mouse femur (Fig 9A, left) showed that variant 5.6 did indeed reach the bones. The observed accumulation of the unconjugated dye in the liver but not in the kidneys or in the bones served to confirm the specificity of variant 5.6 for bone tissue.

### M-CSF_RGD_ variants 4.22 and 5.6 inhibit osteoclast activity in an animal model of osteoporosis

To determine whether variants 4.22 and 5.6 (which gave better production yields than 4.24) inhibit osteoclast activity *in vivo*, we used the well-established mouse model of ovariectomy-induced bone loss (OVX). Two weeks after the mice had undergone ovariectomy or a sham operation, they were injected s.c. twice a day for 3 d with phosphate-buffered saline (PBS), variant 4.22, or variant 5.6. After the last injection, blood was collected, and serum concentrations of the marker for bone resorption (osteoclast activity), the cross-linked carboxy-terminal telopeptide of type I collagen (CTX-I), were determined using ELISA. Two weeks after the operation, there was a significant increase in serum CTX-I levels in ovariectomized mice injected with PBS as compared to the sham-operated mice. Analysis of the CTX-I levels in mice injected with variants 4.22 and 5.6 showed a significant reduction compared to ovariectomized mice injected with vehicle. Strikingly, serum CTX-I levels of mice that were injected with variant 5.6 were reduced almost to the level of CTX-I serum levels of the sham-operated mice (Fig 9B).

## Discussion

A number of studies have demonstrated the crosstalk between biological processes mediated by c-FMS, α_v_β_3_ integrin, and their ligands in the context of osteoclast differentiation, cytoskeleton organization, and bone resorption [14, 16, 19]. Since enhanced osteoclast function is an underlying cause of many bone-related disorders, with osteoclast differentiation and function being dependent on c-FMS and α_v_β_3_ integrin, these two proteins constitute attractive targets for therapeutic intervention. However, since these two receptors regulate common pathways, combination therapy or high drug doses may be needed to fully block the differentiation and resorption processes, with resulting increases in potential side effects and in the cost of therapy. Dual-specific proteins that could bind to and inhibit both c-FMS and α_v_β_3_ integrin would therefore have superior therapeutic potential, as such proteins would offer improved binding avidity and selectivity compared to monospecific protein therapeutics. Nevertheless, despite extensive efforts in the field, there are currently no clinically approved therapeutic agents that are specific for both c-FMS and α_v_β_3_ integrin. The new approach described here, which entails engineering dual-specific therapeutic proteins, is the first to use a natural ligand as a scaffold for engineering high-affinity binding to two therapeutic targets and to apply the engineered bispecific ligands in preclinical studies of osteoporosis.

We have shown in this study that M-CSF is an excellent scaffold for engineering bispecific ligands with affinity and specificity for both c-FMS and α_v_β_3_ integrin. Many integrins recognize the RGD motif in extracellular matrix ligands, including fibronectin, vitronectin, fibrinogen, and osteopontin [38]. Since the RGD motif in natural ligands is typically found in flexible solvent-exposed loops, we mutated either loop 1 or loop 3, both of which are located in the dimerization interface of M-CSF_WT_ and remain accessible in M-CSF_RGD_ monomers [39]. Mutating either one of these loops with an RGD sequence flanked by three residues on each side allows M-CSF_RGD_ to interact with α_v_β_3_ integrin. This interaction does not disrupt the contact with the native c-FMS receptor, as the latter interacts with residues located on the opposite side of the M-CSF_RGD_ ligand. Overall, the interaction area between M-CSF_RGD_ variant 4.22 and α_v_β_3_ integrin is relatively large and scattered, in accordance with the physiological integrin–ligand interaction [40].

The M-CSF_RGD_ variants 4.22, 4.24, and 5.6 had enhanced inhibitory capabilities compared to M-CSF_c-FMS_ and M-CSF_αvβ3_, thereby highlighting the effects of the dual functionality that we engineered into these proteins. The M-CSF_RGD_ variants were shown to be better inhibitors of osteoclast actin ring formation and osteoclast differentiation than M-CSF_c-FMS_ and M-CSF_αvβ3_.We showed by SPR and cell-surface–binding assays that the engineered dual-specific M-CSF proteins can bind both α_v_β_3_ integrin and c-FMS receptors, leading to antagonism of immediate signaling events (c-FMS and Akt phosphorylation) and downstream biochemical events regulating osteoclast differentiation. Moreover, the presence of α_v_β_3_ integrin and c-FMS on the surface of the cell enhances M-CSF_RGD_’s capability to bind it and lead to a stronger inhibition than the monospecific controls.

The three M-CSF_RGD_ proteins, 5.6, 4.22, and 4.24, which differed in the amino acids flanking the RGD motif, also differed in their binding affinities for c-FMS and α_v_β_3_ integrin. These variations in affinities were translated into differences in their biological activities. Variant 5.6 had the highest binding affinity to c-FMS and accordingly showed the highest binding and inhibition of c-FMS and Akt phosphorylation in murine pre-osteoclast cells and the lowest inhibitory effect on actin ring formation. However, variant 5.6 exhibited lower inhibition of murine osteoclast differentiation *in vitro* than variants 4.22 and 4.24. The latter two variants showed higher binding affinity to α_v_β_3_ integrin and a drastic inhibition of actin ring formation, which may suggest that increased binding to integrin led to a more efficient inhibition of murine osteoclast differentiation *in vitro*. This notion is supported by our observation that inhibition of murine osteoclast differentiation was higher for M-CSF_αvβ3_ (the α_v_β_3_-integrin–only binder) than for variant 5.6. In contrast, variant 5.6 showed higher efficiency than M-CSF_αvβ3_ in inhibiting human osteoclast differentiation *in vitro*. Moreover, variant 5.6 showed better inhibition of bone resorption *in vivo* than variant 4.22, which suggests that higher affinity to c-FMS results in stronger inhibition of human osteoclast differentiation *in vitro* and promotes inhibition of osteoclast activity in mice *in vivo*. These results could be explained by the fact that variant 5.6 exhibits better thermal stability than variant 4.22, although we cannot rule out the possibility that other differences, such as differences in proteostability, might affect their activities *in vivo*.

The main goal of this study was to exploit the attributes of M-CSF to engineer a versatile platform for the development of a targeted osteoporosis therapy. By demonstrating that this natural ligand could be engineered to function as a scaffold with the ability to target two receptors at the same time, we showed that engineering of natural agonists to function as receptor antagonists could become an alternative strategy for generating therapeutics not only for osteoporosis but also for other important biomedical targets. We envision that protein variants generated from such efforts could promote the development of the next generation of therapeutics, including, but not limited to, targeted drug delivery agents and selective tissue targeting probes. Similarly, this approach will provide new tools for studying molecular mechanisms and cell signaling pathways—not only those that mediate osteoclast differentiation and function but also pathways that are involved in different pathological processes.

## Methods

### Ethics statement

This study was carried out according to protocols approved by the Ben-Gurion University Committee for the Ethical Care and Use of Animals in Experiments (permit number: IL32072017). All surgery was performed under anesthesia, and all efforts were made to minimize discomfort, distress, and suffering.

### Flow cytometry sorting and analysis

For expressing the M-CSF_C31S_ gene and the two M-CSF_RGD_ libraries on the yeast surface, the yeast culture was grown in the tryptophan-selective SDCAA medium (2% dextrose, 0.67% yeast nitrogen base, 0.5% bacto casamino acids, 1.47% sodium citrate, 0.429% citric acid monohydrate, pH 4.5). This was followed by suspension in SGCAA medium (2% galactose, 0.67% yeast nitrogen base, 0.5% bacto casamino acids, 1.47% sodium citrate, 0.429% citric acid monohydrate) for overnight incubation at 30 °C until the culture reached OD_600_ = 10.0 (10^8^ cells per ml). A number of cells equal to 10 times the library size was added to 1 ml PBS containing 1% bovine serum albumin (PBSA); the suspension was centrifuged, and the supernatant was removed. For analysis of binding of M-CSF_C31S_ and the M-CSF_RGD_ libraries to c-FMS, yeast cells were incubated with mouse anti-c-myc antibody, 9E10 (Abcam, Cambridge, MA, USA) in a 1:50 ratio and different concentrations of soluble Fc-conjugated human c-FMS (R&D systems, Minneapolis, MN, USA) for 1 h at room temperature. After an additional washing step, cells were labeled with secondary phycoerythrin (PE) anti-mouse (Sigma-Aldrich, St. Louis, MO, USA), and goat anti-human Fc FITC antibodies (Sigma-Aldrich) for 30 min at 4 °C in the dark. For analysis of binding of M-CSF_RGD_ libraries to integrin, the experiment was performed in integrin binding buffer (IBB; 20 mM Tris, pH 7.5, 2 mM CaCl_2_, 100 mM NaCl, 1 mM MgCl_2_, and 1 mM MnCl_2_) + 1% BSA. The yeast cells were incubated with chicken anti-c-myc and different concentrations of soluble human α_v_β_3_ integrin (R&D Systems) for 1 h. After an additional washing step, cells were labeled with secondary PE-anti-chicken and FITC-anti-human β_3_ integrin (BioLegend, San Diego, CA, USA) antibodies for 30 min in the dark. Labeled cells were washed with 1 ml PBSA or IBB + 1% BSA. A total of five rounds of affinity maturation sorting were performed; in each sort, a diagonal shape was created with the aim to enrich the high protein binders. In each sort, 0.5% to 2% of the population was collected into a new tube containing SDCC medium. The first sort was performed against 500 nM c-FMS (sort 0), and the subsequent sorts against 500 nM α_v_β_3_ integrin (sort 1), 250 nM α_v_β_3_ integrin (sort 2), 100 nM α_v_β_3_ integrin (sort 3), 20 nM α_v_β_3_ integrin (sort 4), and 50 nM of c-FMS (sort 5). The high-affinity binders were enriched using an iCyt Synergy FACS apparatus (Sony Biotechnology, San Jose, CA, USA).

### Choosing and identifying high-affinity α_v_β_3_ integrin and c-FMS binders

To identify the best α_v_β_3_ integrin and c-FMS binders, 25 different clones from sort 4 and 25 different clones from sort 5 were tested for binding to 20 nM α_v_β_3_ integrin, as described in the flow-cytometry–sorting section. Then, 10 α_v_β_3_ integrin binding clones from sort 4 and 10 α_v_β_3_ integrin binding clones from sort 5 having the highest affinities to α_v_β_3_ integrin were analyzed for binding to 50 nM c-FMS. The clones with the highest binding affinity for both α_v_β_3_ integrin and c-FMS were selected, and DNA was extracted from these clones using Zymoprep Yeast Plasmid Miniprep I (Zymo Research, Irvine, CA, USA) according to the manufacturer's protocol. The extracted plasmids were incubated with *Escherichia coli*-competent cells for 30 min on ice and transferred into 0.2-cm gap cuvettes (Bio-Rad, Hercules, CA, USA). The cuvettes were inserted into a Micropulser electroporator (Bio-Rad) and pulsed with 2.5 kV. Immediately, 1 ml warm Luria Broth (LB) medium was added to each cuvette, and the suspensions were incubated at 37 °C for 1 h. The bacteria were seeded on LB agar plates containing 1:1,000 ampicillin and grown overnight at 37 °C. Colonies were moved to LB medium containing ampicillin and grown overnight. The plasmids were extracted from the bacterial culture with HiYield plasmid mini kit (RBC, Bioscience, Taiwan) according to the manufacturer's protocol. The purified plasmids were sequenced to confirm that they contained the desired sequence and to prevent repetitions (The DNA Microarrays and DNA Sequencing Laboratory, National Institute for Biotechnology in the Negev, Ben-Gurion University of the Negev [NIBN, BGU], Beer-Sheva, Israel). To determine the binding specificity to other RGD binding integrins—namely, α_v_β_5,_ α_4_β_7_, α_2_β_2b_, and α_5_β_1_ (BioLegend)—the three chosen M-CSF_RGD_ variants (4.22, 4.24, and 5.6) were analyzed for binding to 250 nM of each integrin. To detect integrin binding, M-CSF_RGD_ variants were incubated with APC anti-human CD49d, APC anti-human CD41, FITC anti-human CD49e, or FITC anti-human CD5 (BioLegend) and analyzed with Accuri C6 flow cytometry analyzer (BD Biosciences, San Jose, CA, USA). M-CSF_c-FMS_, M-CSF_αvβ3_, and M-CSF_RGD_ variants were produced, purified, and characterized as described in S1 Text.

### SPR

Determination of binding of the soluble purified proteins to c-FMS and α_v_β_3_ integrin was performed on a ProteOn XPR36 (Bio-Rad). The M-CSF_RGD_ variants, M-CSF_c-FMS_, and M-CSF_αvβ3_ proteins were immobilized on the surface of the chip by using the amine coupling reagents sulfo-NHS, 0.1 M, and EDC (1-ethyl-3-[3dimethylaminopropyl]-carbodiimide, Bio-Rad), 0.4 M. To attach the M-CSF_RGD,_ M-CSF_c-FMS_, and M-CSF_αvβ3_ variants covalently to the chip, 1 μg of each protein and 3 μg of BSA in 10 mM sodium acetate buffer (pH 4.0) were used to give 1,141, 900, 1,329, 1,272, and 1,337 response units (RUs) for M-CSF_c-FMS_ and M-CSF_αvβ3_ variants 4.22, 4.24, and 5.6, respectively. Unbound esters were deactivated with 1 M ethanolamine HCl at pH 8.5, and the temperature was set at 25 °C. Then, the chip was rotated, and the human c-FMS soluble proteins (Sino Biological, China) were allowed to flow over the chip at six different concentrations (0, 12.5, 25, 50, 100, and 200 nM) at a flow rate of 50 μl/min for 490 s, followed by dissociation for 600 s in PBS + 0.005% Tween (PBST). For determining α_v_β_3_ integrin binding, the chip was regenerated using 50 mM NaOH at a flow rate of 100 μl/min, and different concentrations of α_v_β_3_ integrin proteins (0, 12.5, 25, 50, 100, and 200 nM) were allowed to flow over the chip. The interactions obtained were normalized to the initial protein binding RUs to the chip surface. Then, for each concentration, the K_D_ was obtained from the equilibrium binding phase of the sensorgram. To determine the α_v_β_3_ integrin specificity for each M-CSF_RGD_ variant, a new chip was loaded with different RGD binding integrins, as follows: 8.5 μg of α_3_β_1_ integrin, 8.5 μg of α_4_β_7_ integrin, 6 μg of α_5_β_1_ integrin, and 8.5 μg of α_v_β_3_ integrin, as well as 3 μg of BSA as a negative control. The proteins were covalently bound to the chip with 10 mM sodium acetate buffer (pH 4.0) as described above. Then, 1 μM of the M-CSF_RGD_ variant was allowed to flow over the chip at a rate of 50 μl/min for 409 s, followed by 600 s of dissociation with PBS + 1% Tween. The interactions obtained were normalized to the initial protein binding RUs to the chip surface. To achieve statistical significance, we made sure that the χ^2^ values were at least 10% or lower than the R_max_ values.

### Computational docking model of α_v_β_3_ integrin, M-CSF_RGD_, and c-FMS complex

Molecular coordinates for the α_v_β_3_ integrin headpiece were taken from the 1L5G Protein Data Bank (PDB) structure [29] (1–438 of the α_v_ subunit and 55–432 of the β_3_ subunit). M-CSF_WT_ in complex with c-FMS was obtained from the 4WRL PDB structure [39]. The M-CSF_RGD_ variant 4.22 was created by replacing residues 25–32 of the native protein with the residues QTSRGDSPS by using PyMOL Molecular Graphics System, Version 1.8 Schrödinger, LLC (De Lano). The M-CSF_RGD_ structure was energy minimized using the Gromacs 4.6.7 package of programs [41]. Next, M-CSF_RGD_ together with the α_v_β_3_ integrin headpiece were subjected to a receptor-ligand docking procedure by using the PatchDock server [28]. To avoid irrelevant structures, potential binding sites both for the α_v_β_3_ integrin receptor and the M-CSF_RGD_ ligand were defined according to PatchDock recommendations. Slight variations in the interaction restraints yielded five experiments, thus resulting in a total of 500 structures. These were clustered with a 0.5-nm cutoff by using Gromacs. The c-FMS receptor was aligned to the representative models, and snapshots were prepared by the VMD program [42].

### Cell binding assay

M-CSF_c-FMS_, M-CSF_αvβ3_, and two variants of M-CSF_RGD_ (4.22 and 5.6) were labeled with a molar ratio of 1:1 DyLight 488 NHS Ester (Thermo Fisher Scientific) and the purified protein. The solution was incubated for 1 h at room temperature, and the residual unbound dye was washed three times with Vivaspin (GE Healthcare Life Sciences) with a 3,000-Da cutoff. M-CSF_RGD_ variant 4.24 was not tested for direct cell binding because of low protein-purification yields. The following two assays were conducted:

- MDA-MB-231 breast cancer cells (ATCC HTB-28) were used for assaying direct cell binding of the purified proteins. Cells were plated at a density of 10^5^ per well in 96-well plates and washed with 1 ml 0.1% PBSA. Cells were then centrifuged at 150g for 5 min, and the supernatant was removed; this step was repeated twice. Then, labeled M-CSF_c-FMS_, M-CSF_αvβ3_ or M-CSF_RGD_ protein variants were added in IBB + 1% BSA to the cells at different concentrations (1, 2.5, and 7.5 μM) in a total volume of 100 μl and incubated at 4 °C with gentle agitation for 2 h. For the competition assay for variant 5.6, the cells were incubated with 1 μM 5.6 in the presence of 5 μM M-CSF_WT_ or 10 μM cRGD separately or with both together. For determination of c-FMS expression, the cells were stained with PE-anti human CD115 (BioLegend) at a dilution of 1:50, and for α_v_β_3_ integrin expression, with FITC-anti human β_3_ integrin (BioLegend) at a dilution of 1:25 for 30 min. Then, the cells were washed twice as described above and analyzed with an Accuri C6 flow cytometry analyzer (BD Biosciences). The mean autofluorescence of the cells was subtracted from the fluorescence reads of all samples. The experiment was repeated three times at each concentration.
- Murine BMMs were obtained by killing mice with isoflurane (Piramal, India), followed by flushing the bone marrow from the femurs and tibias of WT C57BL6 mice. The cells were treated with ACK red lysis buffer (Thermo Fisher Scientific) and plated on bacterial culture dishes in αMEM growth medium (Sigma-Aldrich) containing recombinant murine M-CSF (40 ng/ml) (R&D systems). The cells were incubated for 3 d at 37 °C under 5% CO_2_ to induce monocyte adhesion and proliferation. The plates were washed with PBS, the cells were detached using a cell scraper, and 10^5^ cells were transferred to each well of a 96-well plate. The cells were washed twice with 200 μl 0.1% PBSA. Binding was determined as described for labeled M-CSF_RGD_ proteins. For determination of murine c-FMS expression, anti-mouse CD115 (CSF-1R) (BioLegend) was used at 1:50 concentration and detected with goat anti-rat (PE) antibody (Abcam). For determination of α_v_β_3_ integrin expression, Alexa Fluor 488 anti-mouse/rat CD61 antibody (BioLegend) was used as previously described. Samples were analyzed with an Accuri C6 flow cytometry analyzer (BD Biosciences). The experiment was repeated three times at each concentration.

### c-FMS and Akt phosphorylation assay

Murine BMM cells were obtained as described in the previous section. For this assay, 700,000 cells per well were seeded for differentiation with M-CSF (20 ng/ml) and RANKL (20 ng/ml) in a 6-well plate for 48 h held in an incubator at 37 °C. Then, the cells were incubated for 4 h in starvation medium (αMEM growth medium without fetal bovine serum [FBS]) at 37 °C. For the competition assay, cells were washed with PBS, and 1 μM of M-CSF_RGD_ variants and M-CSF_c-FMS_ with M-CSF_WT_ (20 ng/ml) were added to each well for 1 min for c-FMS and 10 min for Akt; the plates were then immediately placed on ice. Cells were lysed with lysis buffer (deoxycholate 0.5%, 25 mM NaF, 10 mM NaPO_4_, 1 mM Na_3_VO_4_×2H_2_O, 5 mM EDTA, 5 mM EGTA, 100 mM NaCl, 2% Triton X-100, protease inhibitors cocktail, 20 mM para-nitrophenylphosphate [PNPP]) and scraped from the wells, incubated for 10 min on ice, and centrifuged at 14,000 × *g* at 4 °C for 30 min; the supernatants were then transferred to fresh tubes. To determine whether the variants spontaneously activated c-FMS or Akt, the proteins were added without M-CSF_WT_, followed by lysis as described. Protein concentrations were determined using a BCA kit (Thermo Fisher Scientific) according to the manufacturer's protocol. To include and analyze a large sample size, proteins from separate gels were compared using the following method. Fifteen μg of three protein samples were boiled, loaded, and separated on each 10% SDS PAGE for c-FMS and 12% SDS PAGE for Akt (for example, a gel was organized in the following way: protein ladder | 4.22 #1| 4.22 #2 | 4.22 #3 | protein ladder | 4.22 #1 | 4.22 #2 | 4.22 #3, with #1 in both lanes representing the same biological sample, #2 representing the same sample, etc.). Proteins were transferred to a PVDF membrane (Bio-Rad, CA, USA) using a Trans-Blot Turbo Transfer System (Bio-Rad, CA, USA). Membranes were cut into three parts, for example: ladder to 4.22 #3 that was blotted to c-FMS (first 4 samples from left to right above), ladder to 4.22 #3 that was blotted to pc-FMS (phosphorylated form of c-FMS), and low Mw part that was blotted to detect actin. In order to reduce the effects of different exposure times and device errors after blotting that was done with the same antibody stock, all the membranes were imaged at the same day with the same developing system (Fusion FX device manufactured by Vilber Lourmat), the same ECL incubation, and the same exposure times. To make sure that the same amount of protein was loaded and that the ECL-developing process was correct, we quantified and normalized each lane to its corresponding actin levels to see that there were no problems with loading or developing (Densitometry values are included in S2 Data).

Every sample was normalized to its corresponding actin densitometric value (that was run on the same gel in the same lane) to exclude the effect of different exposure times. For example, 4.22 #1 pc-FMS was normalized to expression levels (4.22 #1 c-FMS) and to its own actin levels (4.22 #1 actin). Membranes were blocked with 5% BSA in TBS (20 mM Tris, 150 mM NaCl, pH 7.6) + 0.1% Tween 20 (TBST) for 1 h. For detection of phosphorylated receptors, a 1:500 ratio of anti-phospho-M-CSF receptor (Tyr723) (Cell Signaling, MA, USA) and a 1:1,000 ratio of anti-phospho-Akt (Ser473) (Cell Signaling) antibodies were added to 5% BSA in TBST for overnight incubation at 4 °C. For total receptor expression, a 1:1,000 ratio of anti-M-CSF receptor (Cell Signaling) and anti-Akt (Cell Signaling) antibodies were added to 5% BSA in TBST for overnight incubation at 4 °C. Membranes were washed three times for 10 min with PBST. Then, the HRP-conjugated anti-rabbit secondary antibody (Cell Signaling) was added at a ratio of 1:1,000 for 1 h, and the membranes were washed again as described. All antibodies were used from the same stock for each blot. The membranes were developed using ECL-developing reagent (Biological Industries, Israel) according to the manufacturer's protocol and imaged using a Fusion FX device (Vilber Lourmat, Germany). Each membrane went through the same exposure time, which was according to the antibody that was used. Membranes were stripped with stripping buffer (2% sodium dodecyl sulfate [SDS], 62.5 mM Tris HCl [pH 6.8], 0.8% β-mercaptoethanol) at 50 °C for 30 min, and the procedure was repeated again with actin antibody as a loading control (Cell Signaling). The intensity of the protein bands was measured using ImageJ [43] with the analyze gels tool, and the phosphorylated c-FMS or Akt band intensity was normalized to c-FMS or Akt expression, respectively, and to its corresponding loading control on the same membrane (actin). The experiments with M-CSF_c-FMS_ and M-CSF_RGD_ variants 4.22 and 5.6 were repeated three times, and those with M-CSF_RGD_ variant 4.24 were repeated six times.

### Osteoclast actin-belt–formation assay

Murine BMMs were obtained as described in the cell binding assay section. For this assay, 7,000 cells per well were seeded for differentiation with M-CSF (20 ng/ml) and RANKL (20 ng/ml) in a 384 μClear plate (Greiner Bio-One, Austria) for 60 h in an incubator at 37 °C. Then, the different inhibitors were added in a concentration of 5 μM for an additional 24 h. Cells were fixed with a fixation solution (paraformaldehyde 3%, Triton X-100 10%, glutaraldehyde 8%) for 15 min at 37 °C. Following fixation, the cells were washed with PBS and incubated with cytoskeleton buffer (10 mM MES, 150 mM NaCl, 5 mM EGTA, 5 mM MgCl_2_, 5 mM glucose, 100 μM sodium borohydride, pH 6.1) for 15 min. To stain for actin, the cells were washed and incubated with Acti-stain 670 phalloidin (Cytoskeleton, CO, USA) for 45 min at room temperature. Thereafter, cells were washed and incubated with DAPI (Thermo Fisher Scientific) for an additional 15 min. Actin rings were visualized by Operetta (Perkin Elmer, MA, USA). Each condition was repeated five times, and from each well, 20 pictures were obtained. For actin belts, quantification osteoclasts were defined as cells harboring three or more nuclei and were counted in a double-blind manner, and actin belts were determined above an intensity threshold by ImageJ software.

### Cell viability assay

Mouse mesenchymal stem cells (MSCs) were obtained by flushing the bone marrow from femurs and tibias of 10-weeks-old mice as described in the cell binding assay section. Cells were cultured in MesenCult MSC Basal Medium with 1/5 MSC stimulatory supplements, 50 U/L penicillin-streptomycin, and 10 mg/mL gentamycin and kept in a Hypoxia Incubator Chamber (Thermo Fisher Scientific, Waltham, MA, USA) under 5% oxygen. Then, 7,000 cells were plated in each well of a 96-well plate and held for 48 h in an incubator at 37 °C in the presence of different concentrations of inhibitors (50 nM, 1 μM, and 5 μM). After 48 h, the XTT reagent (Biological Industries, Israel) was added to the cells according to manufacturer's protocol for 2 h at 37 °C. The plate was rocked gently, and the absorbance was measured at 450 nm and 650 nm for background subtraction with a Synergy 2 multidetection microplate reader (Biotek, Winooski, VT, USA). Each condition was repeated three times, and the blank absorbance measurements and the background absorbance were subtracted from the sample's absorbance.

### Cell death assay

Murine BMMs were obtained as described in the cell binding assay section, washed with PBS, and detached with a cell scraper, and 1.5 × 10^5^ cells were plated in each well of a 24-well plate. To induce differentiation, M-CSF (20 ng/μl) and RANKL (20 ng/μl) with 1 μM of M-CSF_c-FMS,_ M-CSF_αvβ3_, and M-CSF_RGD_ variants and GW2580 (Abcam, Cambridge, MA, USA) were added for 48 h. Then, cells were detached with Accutase (Biological industries, Israel), centrifuged at 300 × *g* for 5 min, and washed three times with PBSA 0.1%. PI was added, and cells were analyzed with Accuri C6 flow cytometry analyzer.

### Cell differentiation assay

Murine BMMs were obtained as described in the cell-binding–assay section, washed with PBS, and detached with a cell scraper, and 2 × 10^4^ cells were plated in each well of a 96-well plate. Human peripheral blood CD14^+^ monocytes (PromoCell, Germany) were grown in complete αMEM medium for 5 d in the presence of human M-CSF (20 ng/μl). Then, 1 × 10^4^ cells were transferred to each well of a 96-well plate. Osteoclast differentiation was induced by culturing the cells in alpha Minimum Essential Medium (αMEM) containing 10% of FBS, 50,000 units penicillin, 50 mg streptomycin, 20 ng/ml murine M-CSF (Peprotech, Israel) or human M-CSF (R&D systems, Minneapolis, MN, USA), and 20 ng/ml murine RANKL (R&D Systems). To determine the influence of the M-CSF_RGD_ variants, M-CSF_c-FMS_, and M-CSF_αvβ3_ on the osteoclasts, the proteins were added to the differentiation medium (with M-CSF and RANKL) in three different concentrations (50 nM, 1 μM, and 5 μM for BMMs and 50 nM, 250 nM, and 1 μM for human CD14^+^). After 72–96 h, once the cells had differentiated fully, they were fixed in 4% paraformaldehyde and stained using a TRAP staining kit (Sigma-Aldrich, USA) according to the manufacturer's protocol with additional staining of the nuclei with DAPI. As a positive control, PBS was used instead of the inhibitors, and negative controls comprised cells incubated in differentiation medium without RANKL and inhibitors. Osteoclast parameters were obtained by analysis of 20 images from random areas in each well; the osteoclasts were observed with an Olympus ×83 microscope with an automated stage. For murine osteoclasts differentiation, a total of 2,340 frames were analyzed for 1,581 osteoclasts and 5,313 nuclei; for human CD14^+^, a total of 1,620 frames were analyzed for 477 osteoclasts and 1,897 nuclei. Osteoclasts were defined as TRAP-positive cells harboring three or more nuclei and were counted in a double-blind manner, and the number of nuclei in the osteoclasts and the total osteoclast surface area were determined using ImageJ software. To evaluate the TRAP staining in each well, the plate was measured with a 650 nm Synergy 2 multidetection microplate reader. Three repeats were performed for every condition, and each repeat was normalized to the positive control values.

### qPCR

Murine BMMs were obtained as described in the cell binding assay section, washed with PBS, and detached with a cell scraper, and 7.5 × 10^5^ cells were plated in each well of a 6-well plate. Then, osteoclast differentiation was induced as described in the cell differentiation assay section with and without inhibitors at a concentration of 50 nM. When cells reached full differentiation, they were washed once with PBS and lysed, and the mRNA was isolated using TRIzol reagent (Thermo Fisher Scientific). cDNA was prepared using a cDNA Reverse Transcriptase kit (Applied Biosystems) according to manufacturer’s protocol. For each reaction, 100 ng of cDNA was used with a SYBR Green gene expression assay (Applied Biosystems) and analyzed with a LightCycler 480 II analyzer (Roche) for OSCAR (forward primer: CACCTACTGTTGCTATTACC, reverse primer: GAACCTTCGAAACTGATGAC) and NFATc1 (forward primer: CCAGTTCTACTTGGATGATG, reverse primer: GTAAGTTGGGATTTCTGAGTG). Expression levels of genes were normalized to the HPRT (forward primer: CCCCAAAATGGTTAAGGTTG, reverse primer: AGTACTCATTATAGTCAAGGGC) reference gene using the comparative Ct method (ΔΔ Ct) that was compared to the positive control.

### *In vivo* imaging

M-CSF_RGD_ variant 5.6 was labeled with DyLight 680-NHS Ester and was washed rigorously to remove residual dye molecules. Unconjugated dye was incubated with Tris for 30 min at room temperature. C57BL6 mice were anesthetized in an isoflurane chamber and injected s.c. with 2 nmol of labeled protein or unconjugated dye. Then, the mice were killed with isoflurane, and their internal organs were removed and placed on the IVIS platform and imaged with the IVIS system (Perkin Elmer, MA, USA). Similarly, 3 h after injection, C57BL6 mice were killed, and their bones were placed on the IVIS platform and photographed.

### Injection of ovariectomized mice with M-CSF_RGD_

Ten-weeks-old female C57BL6 mice were ovariectomized under complete anesthesia. Two weeks after the operation, the mice were injected s.c. twice a day for 3 d with PBS or 10 mg/kg of M-CSF_RGD_ variants 4.22 or 5.6. Three h after the last injection, the mice were anesthetized with isoflurane, and their blood was collected through a cardiac puncture. Then, blood samples were centrifuged at 4,000 × *g* for 10 min, and the serum samples were transferred to fresh tubes. To determine the serum CTX-I levels, a RatLaps ELISA (Immunodiagnostic systems, UK) was used according to manufacturer’s protocol. Hemolytic samples were not used.

### Statistical analysis

The data from the differentiation assay was analyzed for column statistics with GraphPad Prism version 5.00 for Windows (La Jolla, CA, USA). Data is shown as means ± SEM. Statistical significance was determined by column statistics and ANOVA test analysis. A *p* value < 0.05 was considered statistically significant.

## Supporting information

S1 Fig Amino acid sequences of M-CSF_WT_, M-CSF_RGD_ libraries, M-CSF_c-FMS_, M-CSF_αvβ3_ and the three M-CSF_RGD_ variants that were chosen after an affinity maturation process. (A) The amino acid sequence of M-CSF with C31, which is required for dimerization, indicated in red. The two flexible loops in the dimerization interface are colored blue (loop 1, residues 25–32) and green (loop 3, residues 64–71). (B) M-CSF_RGD_ library 1 colored in blue, where residues 25–32 were replaced with an RGD motif having three random amino acids on each side. (C) M-CSF_RGD_ library 2 colored green, where residues 64–71 were replaced with an RGD motif with three random amino acids on each side and C31 was replaced with serine to inhibit disulfide-linked homodimerization. (D) M-CSF_αvβ3_ is based on the sequence of M-CSF_RGD_ variant 4.22 with two single-point mutations in H9A and H15A, indicated in red, to inhibit binding to c-FMS. (E) M-CSF_c-FMS_ was created by changing the RGD motif on M-CSF_RGD_ variant 4.22 to RDG with the aim to prevent binding to α_v_β_3_ integrin. (F) Sequences of the mutated loop of the three M-CSF_RGD_ clones that were selected after four (4.22 and 4.24) and five (5.6) rounds of the affinity maturation process. M-CSF, macrophage colony-stimulating factor; RGD, Arginine-Glycine-Aspartic acid; WT, wild type.
(TIF)

S2 Fig Compatibility of YSD with M-CSF_C31S_. YSD M-CSF_C31S_ was analyzed for (A) forward scatter and side scatter and (B) expression using mouse anti-c-myc antibody followed by a secondary PE-labeled anti mouse antibody. (C) The binding of YSD M-CSF_C31S_ to soluble c-FMS-Fc was detected by a goat anti-human Fc-FITC antibody. (D) Cells expressing M-CSF_C31S_ on the yeast cell wall were incubated with 10 different concentrations of c-FMS-Fc (0.5–2000 nM) and were tested for binding by flow cytometry. The curve shows a good fit to a single binding-site curve, and the apparent K_D_ is 20 nM. Source data can be found in S7 Data. FITC, fluorescein isothiocyanate; M-CSF, macrophage colony-stimulating factor; PE, phycoerythrin; YSD, yeast surface display.
(TIF)

S3 Fig Scheme of YSD construct. The M-CSF_RGD_ library was covalently linked to Aga1p and the yeast cell wall. Binding for c-FMS was determined with c-FMS-Fc recombinant protein and goat anti-human Fc FITC conjugated secondary antibody, and the expression levels were measured with a mouse anti-c-myc primary antibody and PE anti-mouse secondary antibody. For determination of α_v_β_3_ integrin binding, yeast cells were incubated with recombinant α_v_β_3_ integrin and mouse anti-human CD49d FITC secondary antibody, and the expression levels were measured with chicken anti-c-myc primary antibody and PE goat anti-chicken secondary antibody. FITC, fluorescein isothiocyanate; M-CSF, macrophage colony-stimulating factor; PE, phycoerythrin; RGD, Arginine-Glycine-Aspartic acid; YSD, yeast surface display.
(TIF)

S4 Fig FACS dot plot of M-CSF_RGD_ libraries. M-CSF_RGD_ (A–D) library 1 and (E–H) library 2 were analyzed for (A and E) FSC/SSC, (B and F) expression, (C and G) 100 nM c-FMS binding, and (D and H) 500 nM α_v_β_3_ integrin binding. FACS, fluorescence-activated cell sorting; FSC, forward scatter; M-CSF, macrophage colony-stimulating factor; RGD, Arginine-Glycine-Aspartic acid; SSC, side scatter.
(TIF)

S5 Fig FACS FSC and SSC of affinity maturation process. Yeast-displayed mutant libraries were analyzed, and the living cells population in each sort is represented by a black polygon-shaped gate. The affinity maturation sorting process started with (A) a presorted library followed by (B) sort 1, (C) sort 2, (D) sort 3, (E) sort 4, and (F) sort 5. FACS, fluorescence-activated cell sorting; FSC, forward scatter; SSC, side scatter.
(TIF)

S6 Fig Analysis of individual YSD M-CSF_RGD_ clones selected from sorts 4 and 5 for their binding to c-FMS, α_v_β_3_ integrin and other integrins. Twenty-five different clones from each of sorts 4 (A) and 5 (C) were tested for binding to 20 nM of α_v_β_3_ integrin, normalized to the lowest binder. (B) The best 15 α_v_β_3_ integrin M-CSF_RGD_ binders from sort 4 and the best 10 α_v_β_3_ integrin M-CSF_RGD_ binders from sort 5 (D) were evaluated for binding to 50 nM of c-FMS, normalized to M-CSF_C31S_. The chosen clones (4.22, 4.24, and 5.6) are indicated in blue. (E) Variants 4.22, 4.24, and 5.6 were evaluated for integrin specificity by testing their binding to 250 nM of α_4_β_7_, α_IIb_β_3_, α_v_β_5_, and α_5_β_1_ integrins in comparison with their binding to α_v_β_3_ integrin. Source data can be found in S8 Data. M-CSF, macrophage colony-stimulating factor; RGD, Arginine-Glycine-Aspartic acid; YSD, yeast surface display.
(TIF)

S7 Fig Purification of M-CSF_c-FMS,_ M-CSF_αvβ3_, and M-CSF_RGD_ variants. (A) Size exclusion chromatography of nonglycosylated M-CSF_RGD_ clone 4.22 with high molecular weight standards. Variant 4.22 was eluted at the size of 21 kDa. (B) Mass spectrometry of nonglycosylated variant 5.6. (C) CD spectra of nonglycosylated variant 4.22 (red line), nonglycosylated variant 4.24 (blue line), nonglycosylated variant 5.6 (green line), nonglycosylated M-CSF_c-FMS_ (pink line), and nonglycosylated M-CSF_αvβ3_ (gray lines). (D) Temperature-dependent CD measurements of unfolded proteins determined at 217 nm normalized to fully denatured proteins. (E) SDS-PAGE for all purified proteins: nonglycosylated M-CSF_C31S_ (lane 1), nonglycosylated variant 4.22 (lane 2), nonglycosylated variant 4.24 (lane 3), nonglycosylated variant 5.6 (lane 4), nonglycosylated M-CSF_c-FMS_ (lane 5), and non-glycosylated M-CSF_αvβ3_ (lane 6). Source data can be found in S9 Data. CD, circular dichroism; M-CSF, macrophage colony-stimulating factor; RGD, Arginine-Glycine-Aspartic acid.
(TIF)

S8 Fig Chemical cross-linking of purified protein variants. Dimerization of the purified proteins was determined by using increasing concentrations of BS^3^ cross-linker, denaturation, and analysis on SDS-PAGE. M-CSF_WT_ dimerized at all BS^3^ concentrations, but the three M-CSF_RGD_ variants, M-CSF_c-FMS_, and M-CSF_αvβ3_ did not show any dimerization capability. BS^3^, bis(sulfosuccinimidyl)suberate; M-CSF, macrophage colony-stimulating factor; RGD, Arginine-Glycine-Aspartic acid; WT, wild type.
(TIF)

S9 Fig Steady-state equilibrium analysis. To determine the protein K_D,app,_ the RUs at saturation for each protein concentration were plotted, and a fitted curve was created for (A) c-FMS and (B) α_v_β_3_ integrin. Source data and its analysis can be found in S10 Data. RUs, response units.
(TIF)

S10 Fig Binding specificity of M-CSF_RGD_ variants for RGD-binding integrins. (A) α_v_β_3_, (B) α_3_β_1_, (C) α_4_β_7_, and (D) α_5_β_1_ integrins were immobilized on the surface of the chip. Thereafter, the three M-CSF_RGD_ variants 4.22 (green), 4.24 (blue), and 5.6 (red) were allowed to flow over the surface of the chip at a concentration of 1 μM. Source data can be found in S11 Data. M-CSF, macrophage colony-stimulating factor; RGD, Arginine-Glycine-Aspartic acid.
(TIF)

S11 Fig Docking model of the M-CSF_C31S_/c-FMS–αvβ3 integrin complex. M-CSF_C31S_ is shown in pink, c-FMS in cyan, αv in yellow, and β3 in green. Residues 25–32 of M-CSF_C31S_ are represented in red. M-CSF, macrophage colony-stimulating factor.
(TIF)

S12 Fig **M-CSF**_**RGD**_**/α**_**v**_**β**_**3**_ **integrin interface seen from different angles (A–D).** α_v_ in yellow “surf” presentation, β_3_ in green, M-CSF_RGD_ in pink, and the mutant QTSRGDSPS loop in red. M-CSF, macrophage colony-stimulating factor; RGD, Arginine-Glycine-Aspartic acid.
(TIF)

S13 Fig Superposition of M-CSF_RGD_ with crystallized cRGD from the 1L5G PDB structure [29]. α_v_ in yellow, β_3_ in green, RGD from M-CSF_RGD_ in cyan, and RGD from the crystal in pink. cRGD, cyclic RGD; M-CSF, macrophage colony-stimulating factor; RGD, Arginine-Glycine-Aspartic acid.
(TIF)

S14 Fig Receptor expression levels of MDA-MB-231 and mouse BMMs. The expression levels of c-FMS and α_v_β_3_ integrin were measured using flow cytometry. The red histograms represent the negative control, and the blue histograms represent receptor expression. Mouse BMMs without differentiation cytokines (*t* = 0) express c-FMS (A) and α_v_β_3_ integrin (B). MDA-MB-231 breast cancer cell line express c-FMS (C) and α_v_β_3_ integrin (D). BMM, bone-marrow–derived monocyte; MDA-MB-231, MDA Anderson metastatic breast 231.
(TIF)

S15 Fig Akt and c-FMS phosphorylation without murine M-CSF. Murine BMMs were seeded for differentiation for 48 h, followed by incubation of purified M-CSF_c-FMS_, M-CSF_αvβ3_, and M-CSF_RGD_ variants without the addition of murine M-CSF. Cells were lysed and subjected to SDS-PAGE to test spontaneous activation of (A) c-FMS and (B) Akt. The aspect ratios of the membranes were changed. BMM, bone-marrow–derived monocyte; M-CSF, macrophage colony-stimulating factor; RGD, Arginine-Glycine-Aspartic acid.
(TIF)

S16 Fig Actin ring formation in mature osteoclasts incubated with M-CSF_RGD_ variants. Differentiated murine BMMs were incubated for additional 24 h without (positive control) or with inhibitors (5 μM) followed by fixation and F-actin and nuclei staining. Cells were able to form a solid actin ring (white arrowheads), scattered actin ring [44] (white arrows) or amorphous actin distribution (barbed arrowheads). Pictures are representatives of 35 images acquired from five different wells per sample. BMM, bone-marrow–derived monocyte; M-CSF, macrophage colony-stimulating factor; RGD, Arginine-Glycine-Aspartic acid.
(TIF)

S1 Text Additional materials and methods information. (DOCX)

S1 Table Binding affinities of M-CSF_RGD_ variants to c-FMS and α_v_β_3_ integrin determined by SPR. M-CSF, macrophage colony-stimulating factor; SPR, surface plasmon resonance.
(XLSX)

S1 Data Numerical data for generating SPR binding sensorgrams in Fig 3. SPR, surface plasmon resonance.
(XLSX)

S2 Data Numerical data and densitometry results for generating the graphs in Fig 5. (XLSX)

S3 Data Numerical data of actin belts quantification in Fig 6. (XLSX)

S4 Data Numerical data and analysis of graphs in Fig 7. (XLSX)

S5 Data Numerical data and analysis of graphs in Fig 8. (XLSX)

S6 Data Numerical data of CTX serum levels Fig 9. CTX, carboxy-terminal telopeptide.
(XLSX)

S7 Data Numerical data of YSD in S2 Fig. YSD, yeast surface display.
(XLSX)

S8 Data Numerical data of individual clones’ flow cytometry binding in S6 Fig. (XLSX)

S9 Data Numerical data of the protein purification process in S7 Fig. (XLSX)

S10 Data Numerical data of steady state equilibrium analysis in S9 Fig. (XLSX)

S11 Data Numerical data of different integrins binding using SPR in S10 Fig. SPR, surface plasmon resonance.
(XLSX)

## Abbreviations

αMEM: alpha Minimum Essential Medium
BMM: bone-marrow–derived monocyte
BMSC: bone-marrow–derived mesenchymal stromal cells
BS^3^: bis(sulfosuccinimidyl)suberate
CD: circular dichroism
cRGD: cyclic RGD
CTX-I: carboxy-terminal telopeptide of type I collagen
EDC: 1-ethyl-3-(3dimethylaminopropyl)-carbodiimide
FACS: fluorescence-activated cell sorting
FBS: fetal bovine serum
FDA: Food and Drug Administration
FSC: forward scatter
LB: Luria Broth
M-CSF_WT_: wild-type M-CSF
M-CSF: macrophage colony-stimulating factor
MDA-MB-231: MD Anderson metastatic breast 231
MSC: mesenchymal stem cell
NF-кB: nuclear factor–kappa B
NHS: N-hydroxysuccinimide
OSCAR: osteoclast-associated receptor
OVX: ovariectomy
PBS: phosphate-buffered saline
PBSA: PBS containing 1% bovine serum albumin
PBST: PBS + 0.005% Tween
PE: phychoerythrin
PI: propidium iodide
PNPP: para-nitrophenylphosphate
RANKL: receptor activator of nuclear factor–kappa-B ligand
RGD: Arginine-Glycine-Aspartic acid
RUs: response units
s.c.: subcutaneously
SDS: sodium dodecyl sulfate
SPR: surface plasmon resonance
SSC: side scatter
TRAP: tartrate-resistant acid phosphatase
WT: wild type
XTT: 2,3-bis-(2-methoxy-4-nitro-5-sulfophenyl)-2H-tetrazolium-5-carboxanilide
YSD: yeast surface display
