# Supplementary figures and images for: A dual-specific macrophage colony-stimulating factor antagonist of c-FMS and αvβ3 integrin for osteoporosis therapy
Source: PLoS Biol. 2018 Aug 24;16(8):e2002979. doi: 10.1371/journal.pbio.2002979 (PMC6126843; doi:10.1371/journal.pbio.2002979)

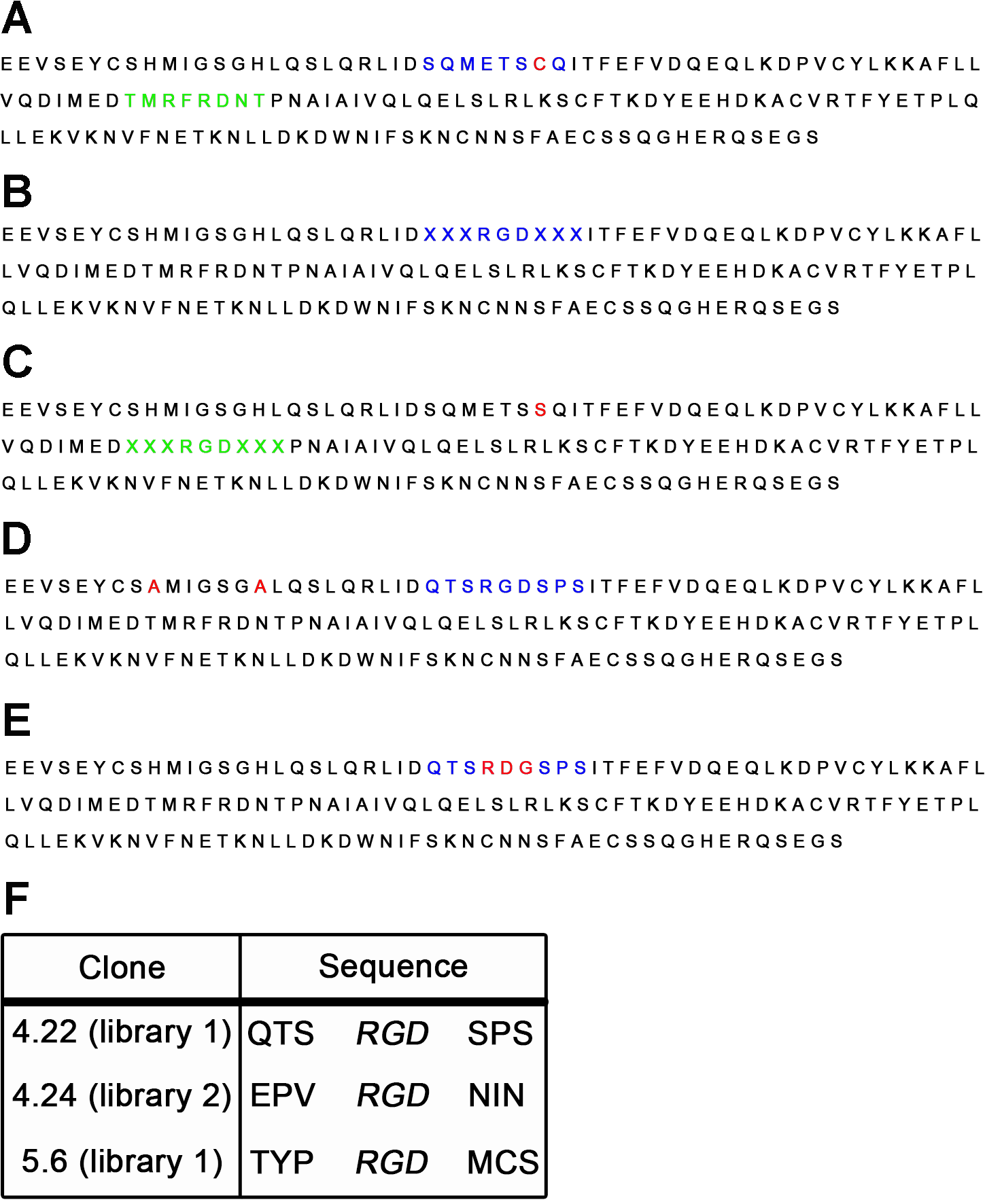

Supplement: S1 Fig — (A) The amino acid sequence of M-CSF with C31, which is required for dimerization, indicated in red. The two flexible loops in the dimerization interface are colored blue (loop 1, residues 25–32) and green (loop 3, residues 64–71). (B) M-CSFRGD library 1 colored in blue, where residues 25–32 were replaced with an RGD motif having three random amino acids on each side. (C) M-CSFRGD library 2 colored green, where residues 64–71 were replaced with an RGD motif with three random amino acids on each side and C31 was replaced with serine to inhibit disulfide-linked homodimerization. (D) M-CSFαvβ3 is based on the sequence of M-CSFRGD variant 4.22 with two single-point mutations in H9A and H15A, indicated in red, to inhibit binding to c-FMS. (E) M-CSFc-FMS was created by changing the RGD motif on M-CSFRGD variant 4.22 to RDG with the aim to prevent binding to αvβ3 integrin. (F) Sequences of the mutated loop of the three M-CSFRGD clones that were selected after four (4.22 and 4.24) and five (5.6) rounds of the affinity maturation process. M-CSF, macrophage colony-stimulating factor; RGD, Arginine-Glycine-Aspartic acid; WT, wild type. (TIF) [file pbio.2002979.s001.tif]

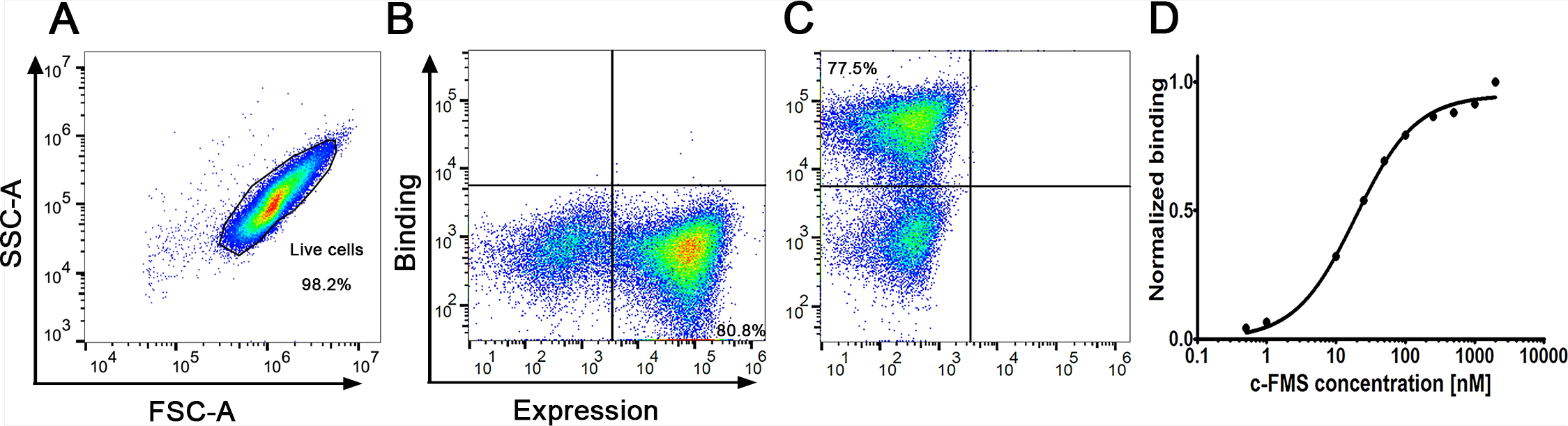

Supplement: S2 Fig — YSD M-CSFC31S was analyzed for (A) forward scatter and side scatter and (B) expression using mouse anti-c-myc antibody followed by a secondary PE-labeled anti mouse antibody. (C) The binding of YSD M-CSFC31S to soluble c-FMS-Fc was detected by a goat anti-human Fc-FITC antibody. (D) Cells expressing M-CSFC31S on the yeast cell wall were incubated with 10 different concentrations of c-FMS-Fc (0.5–2000 nM) and were tested for binding by flow cytometry. The curve shows a good fit to a single binding-site curve, and the apparent KD is 20 nM. Source data can be found in S7 Data. FITC, fluorescein isothiocyanate; M-CSF, macrophage colony-stimulating factor; PE, phycoerythrin; YSD, yeast surface display. (TIF) [file pbio.2002979.s002.tif]

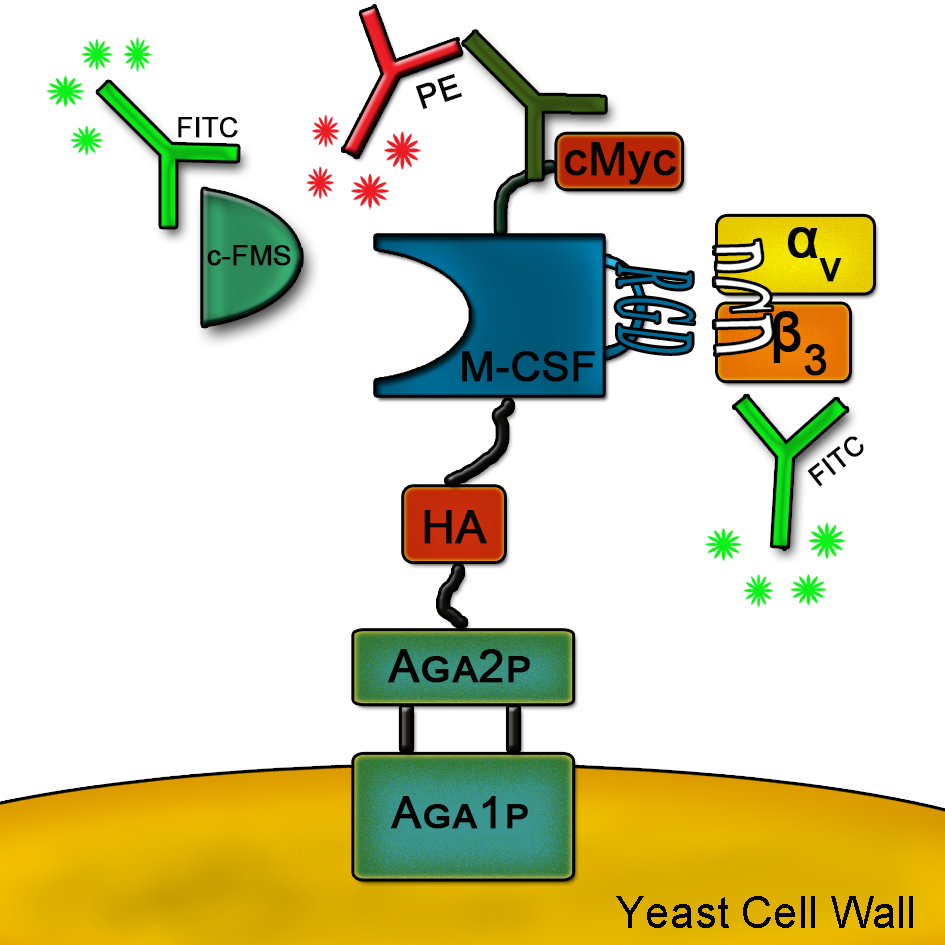

Supplement: S3 Fig — The M-CSFRGD library was covalently linked to Aga1p and the yeast cell wall. Binding for c-FMS was determined with c-FMS-Fc recombinant protein and goat anti-human Fc FITC conjugated secondary antibody, and the expression levels were measured with a mouse anti-c-myc primary antibody and PE anti-mouse secondary antibody. For determination of αvβ3 integrin binding, yeast cells were incubated with recombinant αvβ3 integrin and mouse anti-human CD49d FITC secondary antibody, and the expression levels were measured with chicken anti-c-myc primary antibody and PE goat anti-chicken secondary antibody. FITC, fluorescein isothiocyanate; M-CSF, macrophage colony-stimulating factor; PE, phycoerythrin; RGD, Arginine-Glycine-Aspartic acid; YSD, yeast surface display. (TIF) [file pbio.2002979.s003.tif]

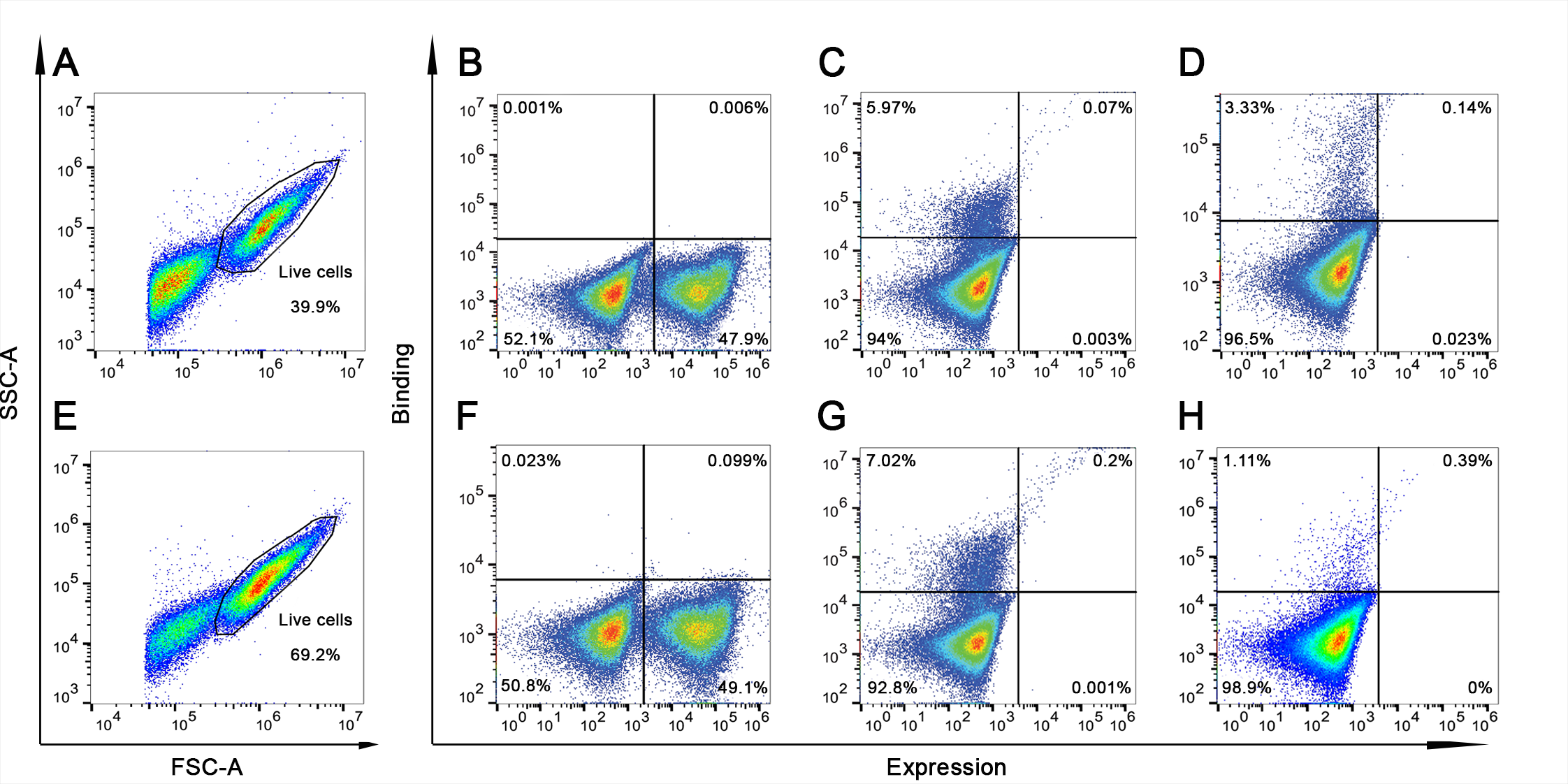

Supplement: S4 Fig — M-CSFRGD (A–D) library 1 and (E–H) library 2 were analyzed for (A and E) FSC/SSC, (B and F) expression, (C and G) 100 nM c-FMS binding, and (D and H) 500 nM αvβ3 integrin binding. FACS, fluorescence-activated cell sorting; FSC, forward scatter; M-CSF, macrophage colony-stimulating factor; RGD, Arginine-Glycine-Aspartic acid; SSC, side scatter. (TIF) [file pbio.2002979.s004.tif]

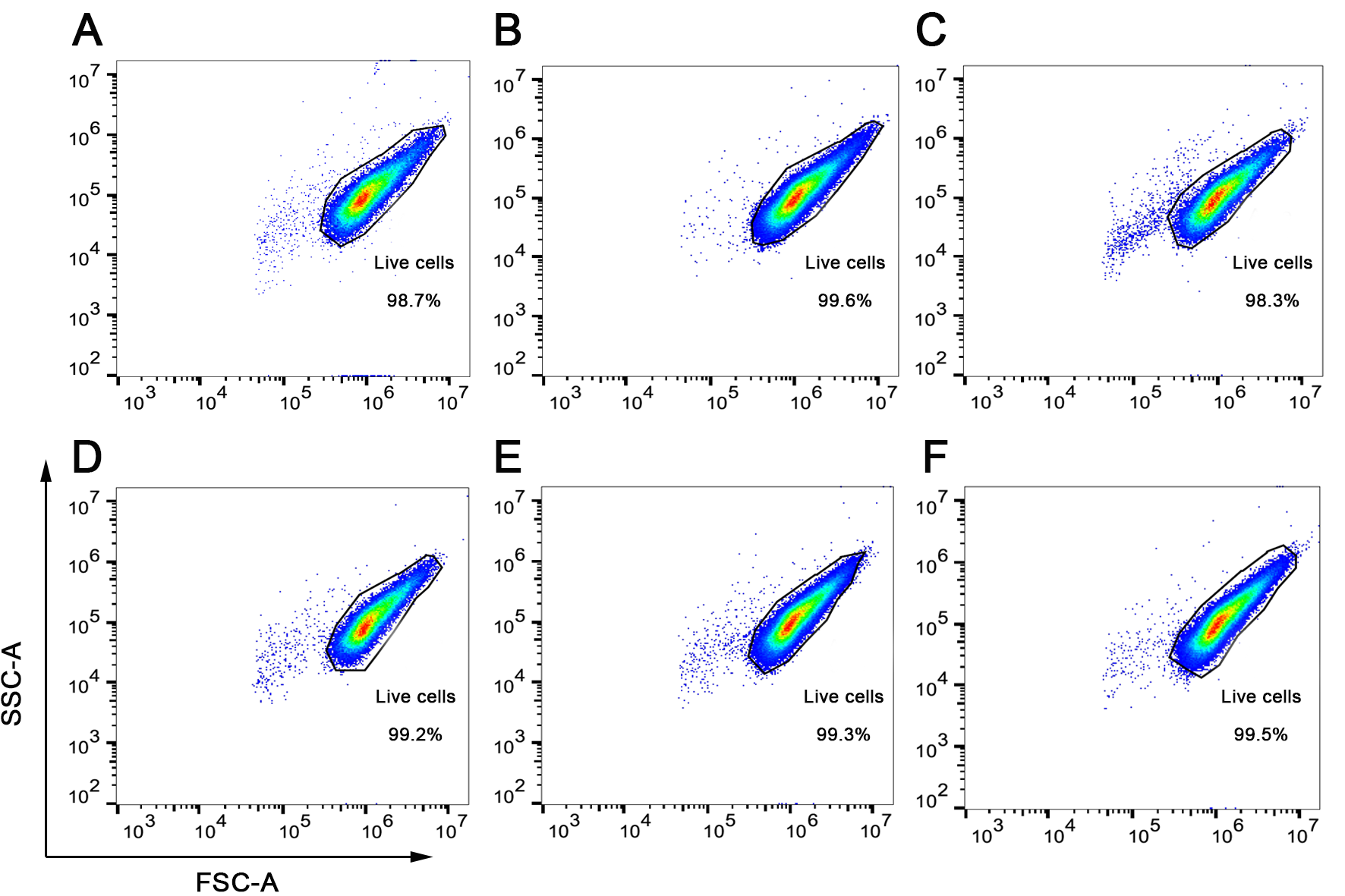

Supplement: S5 Fig — Yeast-displayed mutant libraries were analyzed, and the living cells population in each sort is represented by a black polygon-shaped gate. The affinity maturation sorting process started with (A) a presorted library followed by (B) sort 1, (C) sort 2, (D) sort 3, (E) sort 4, and (F) sort 5. FACS, fluorescence-activated cell sorting; FSC, forward scatter; SSC, side scatter. (TIF) [file pbio.2002979.s005.tif]

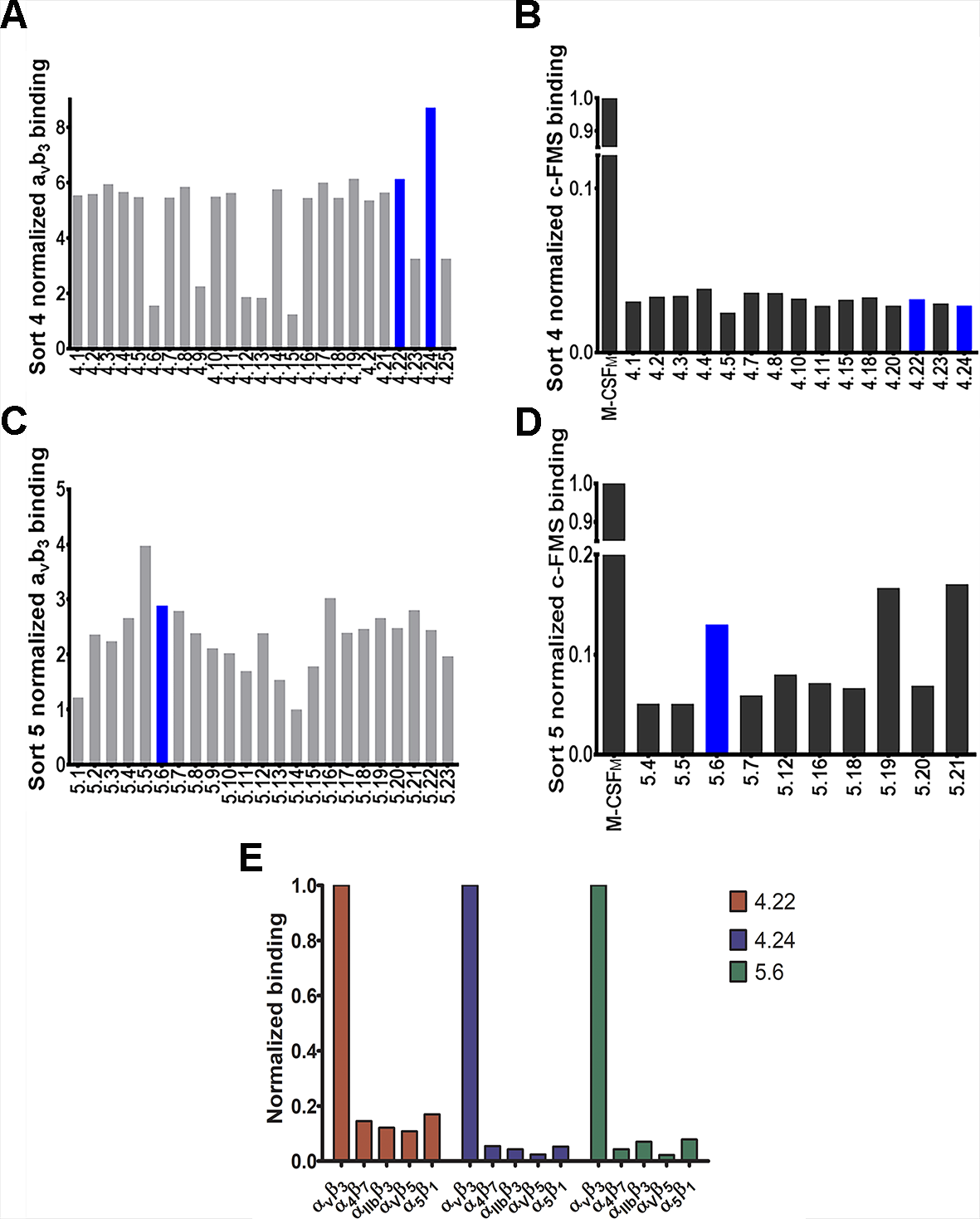

Supplement: S6 Fig — Twenty-five different clones from each of sorts 4 (A) and 5 (C) were tested for binding to 20 nM of αvβ3 integrin, normalized to the lowest binder. (B) The best 15 αvβ3 integrin M-CSFRGD binders from sort 4 and the best 10 αvβ3 integrin M-CSFRGD binders from sort 5 (D) were evaluated for binding to 50 nM of c-FMS, normalized to M-CSFC31S. The chosen clones (4.22, 4.24, and 5.6) are indicated in blue. (E) Variants 4.22, 4.24, and 5.6 were evaluated for integrin specificity by testing their binding to 250 nM of α4β7, αIIbβ3, αvβ5, and α5β1 integrins in comparison with their binding to αvβ3 integrin. Source data can be found in S8 Data. M-CSF, macrophage colony-stimulating factor; RGD, Arginine-Glycine-Aspartic acid; YSD, yeast surface display. (TIF) [file pbio.2002979.s006.tif]

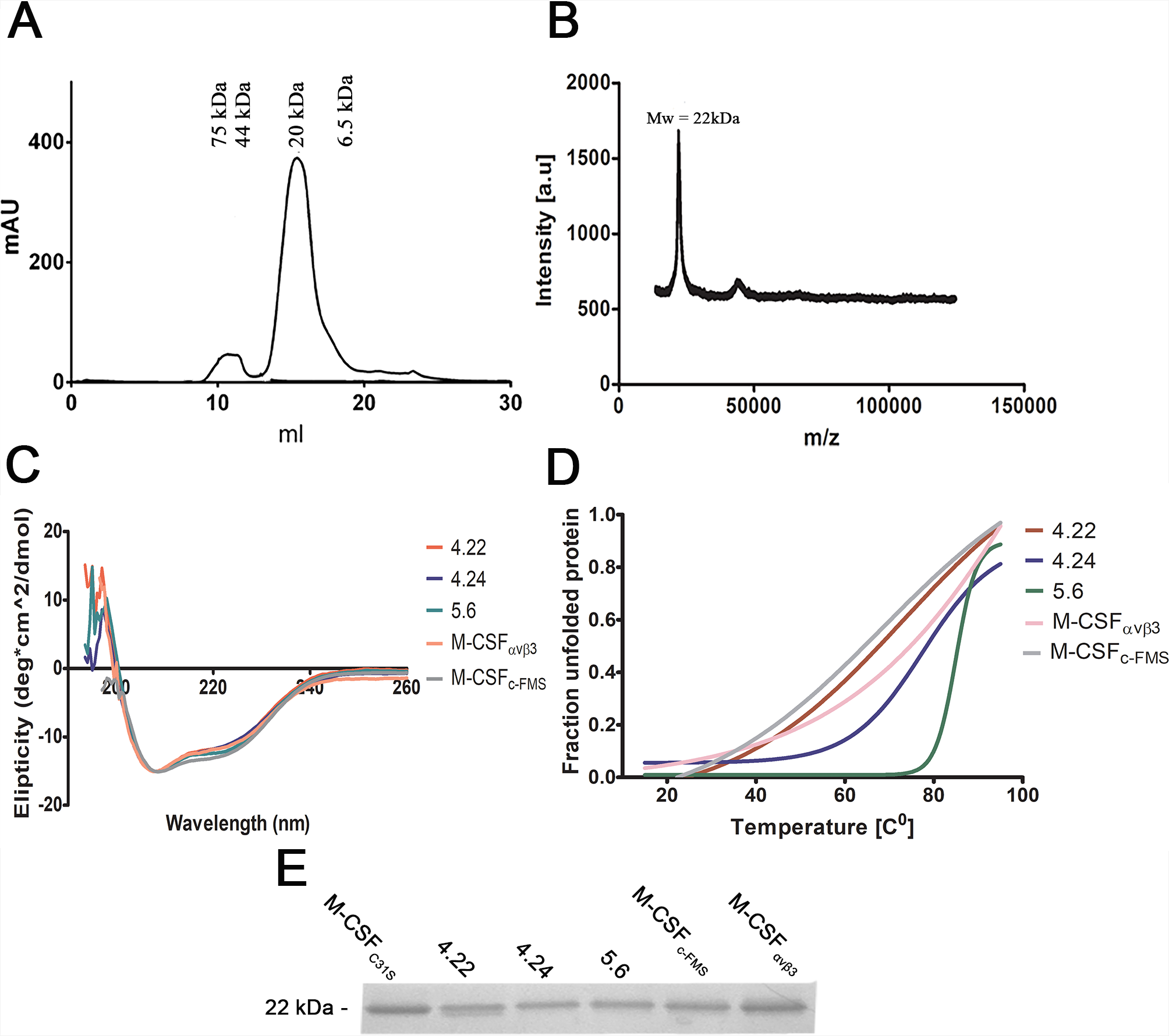

Supplement: S7 Fig — (A) Size exclusion chromatography of nonglycosylated M-CSFRGD clone 4.22 with high molecular weight standards. Variant 4.22 was eluted at the size of 21 kDa. (B) Mass spectrometry of nonglycosylated variant 5.6. (C) CD spectra of nonglycosylated variant 4.22 (red line), nonglycosylated variant 4.24 (blue line), nonglycosylated variant 5.6 (green line), nonglycosylated M-CSFc-FMS (pink line), and nonglycosylated M-CSFαvβ3 (gray lines). (D) Temperature-dependent CD measurements of unfolded proteins determined at 217 nm normalized to fully denatured proteins. (E) SDS-PAGE for all purified proteins: nonglycosylated M-CSFC31S (lane 1), nonglycosylated variant 4.22 (lane 2), nonglycosylated variant 4.24 (lane 3), nonglycosylated variant 5.6 (lane 4), nonglycosylated M-CSFc-FMS (lane 5), and non-glycosylated M-CSFαvβ3 (lane 6). Source data can be found in S9 Data. CD, circular dichroism; M-CSF, macrophage colony-stimulating factor; RGD, Arginine-Glycine-Aspartic acid. (TIF) [file pbio.2002979.s007.tif]

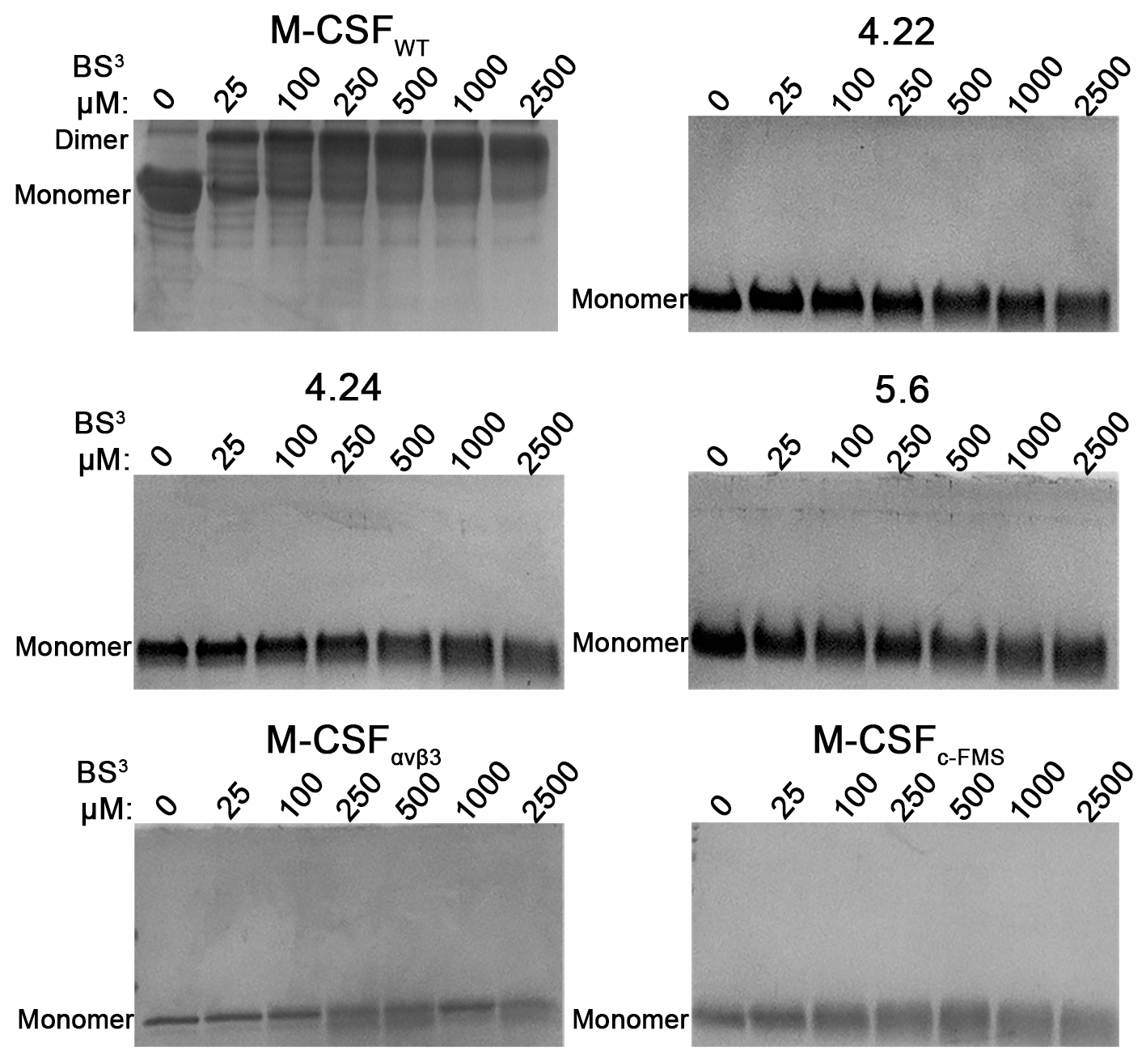

Supplement: S8 Fig — Dimerization of the purified proteins was determined by using increasing concentrations of BS3 cross-linker, denaturation, and analysis on SDS-PAGE. M-CSFWT dimerized at all BS3 concentrations, but the three M-CSFRGD variants, M-CSFc-FMS, and M-CSFαvβ3 did not show any dimerization capability. BS3, bis(sulfosuccinimidyl)suberate; M-CSF, macrophage colony-stimulating factor; RGD, Arginine-Glycine-Aspartic acid; WT, wild type. (TIF) [file pbio.2002979.s008.tif]

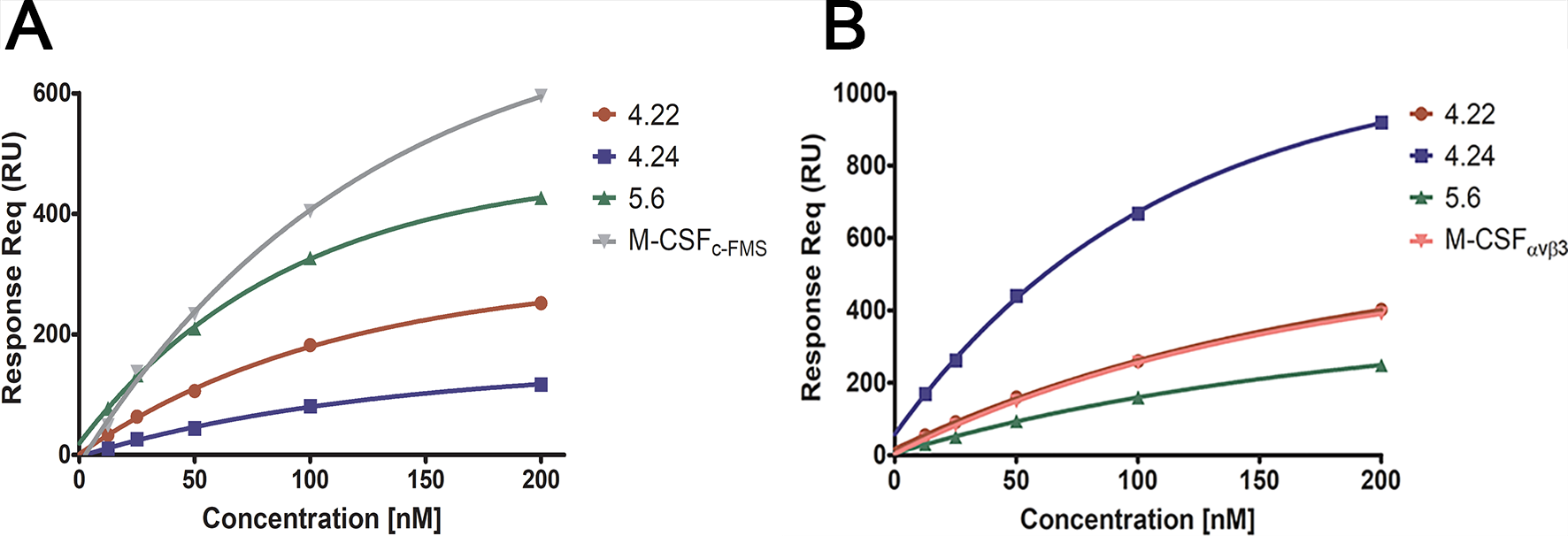

Supplement: S9 Fig — To determine the protein KD,app, the RUs at saturation for each protein concentration were plotted, and a fitted curve was created for (A) c-FMS and (B) αvβ3 integrin. Source data and its analysis can be found in S10 Data. RUs, response units. (TIF) [file pbio.2002979.s009.tif]

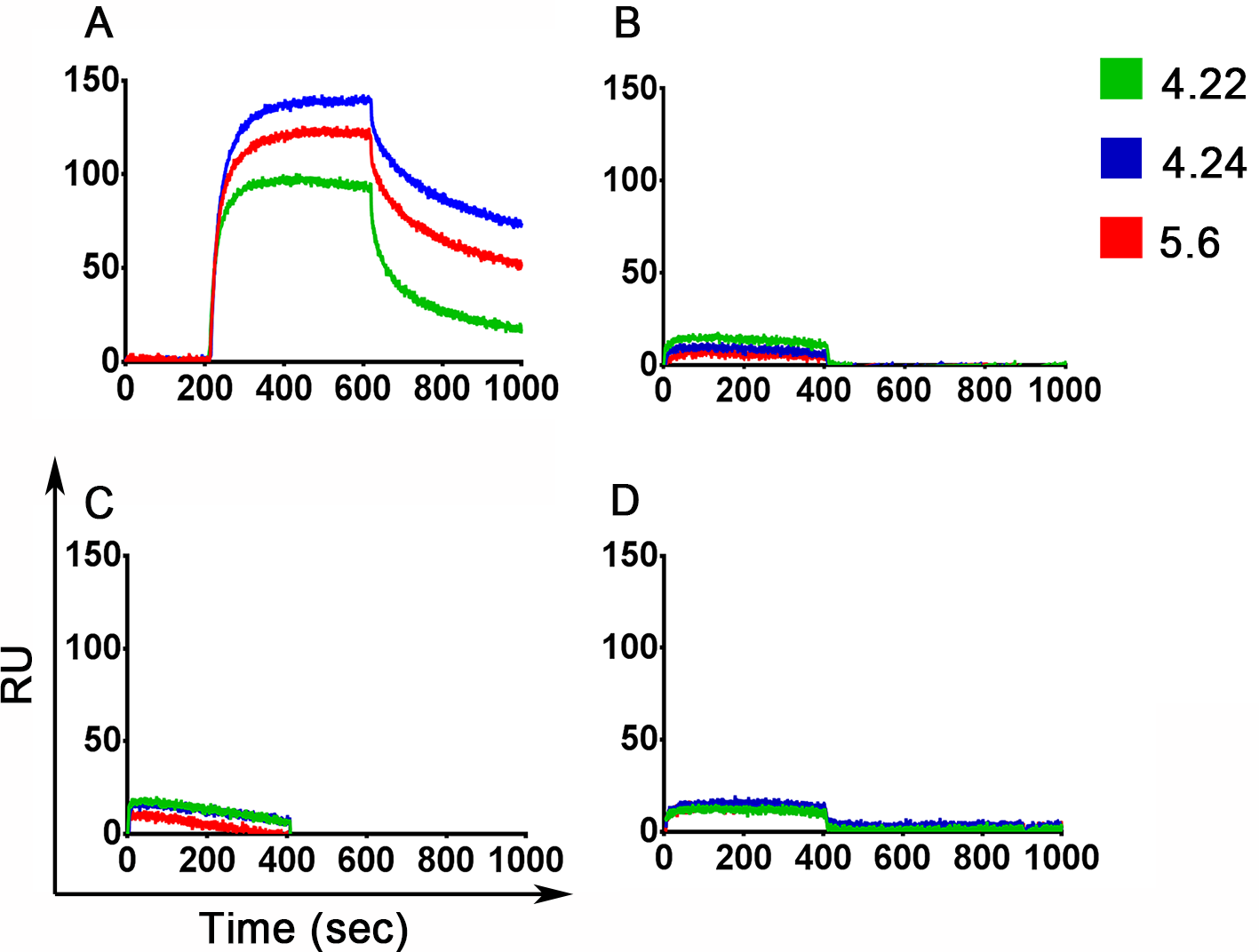

Supplement: S10 Fig — (A) αvβ3, (B) α3β1, (C) α4β7, and (D) α5β1 integrins were immobilized on the surface of the chip. Thereafter, the three M-CSFRGD variants 4.22 (green), 4.24 (blue), and 5.6 (red) were allowed to flow over the surface of the chip at a concentration of 1 μM. Source data can be found in S11 Data. M-CSF, macrophage colony-stimulating factor; RGD, Arginine-Glycine-Aspartic acid. (TIF) [file pbio.2002979.s010.tif]

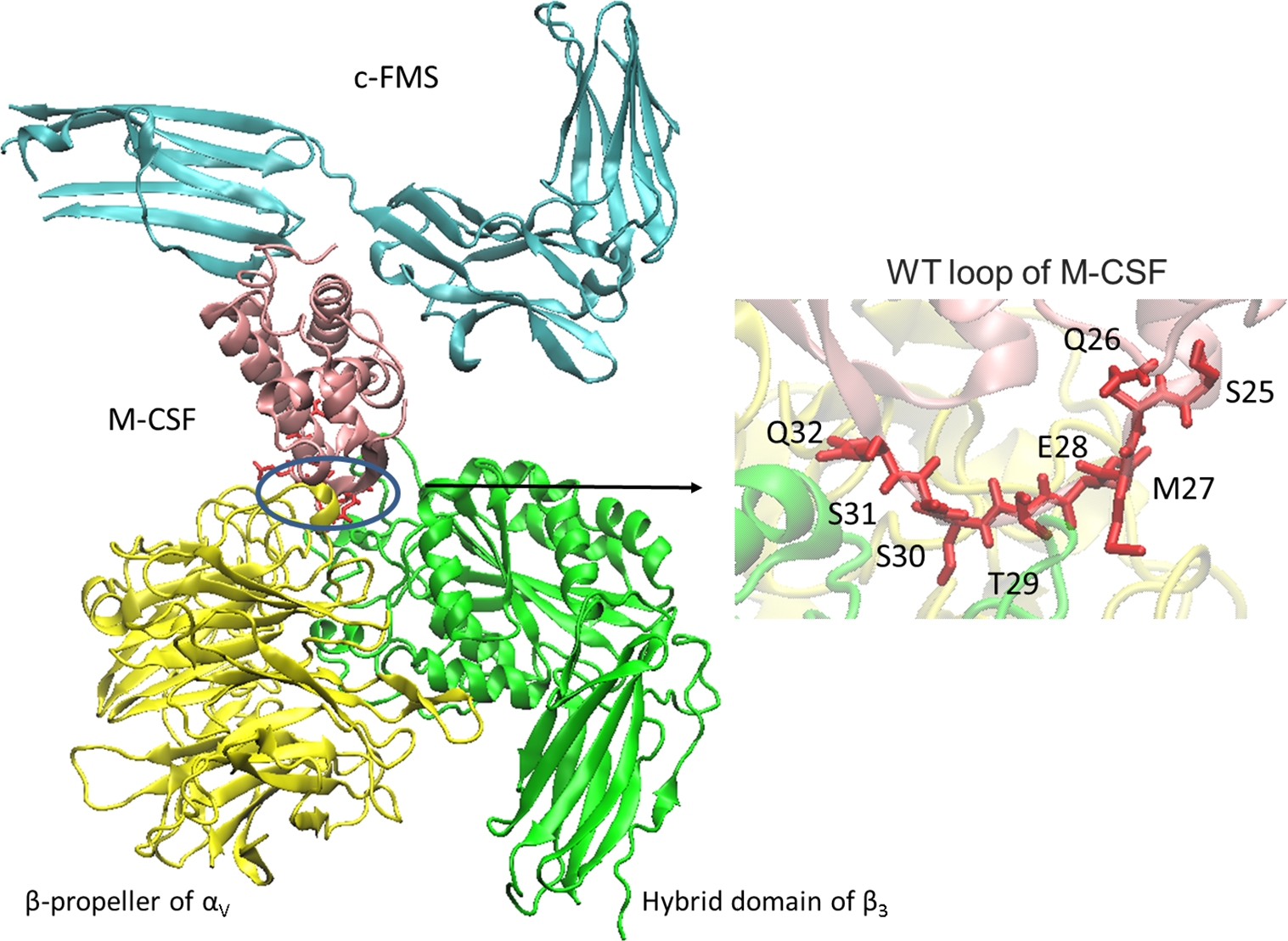

Supplement: S11 Fig — M-CSFC31S is shown in pink, c-FMS in cyan, αv in yellow, and β3 in green. Residues 25–32 of M-CSFC31S are represented in red. M-CSF, macrophage colony-stimulating factor. (TIF) [file pbio.2002979.s011.tif]

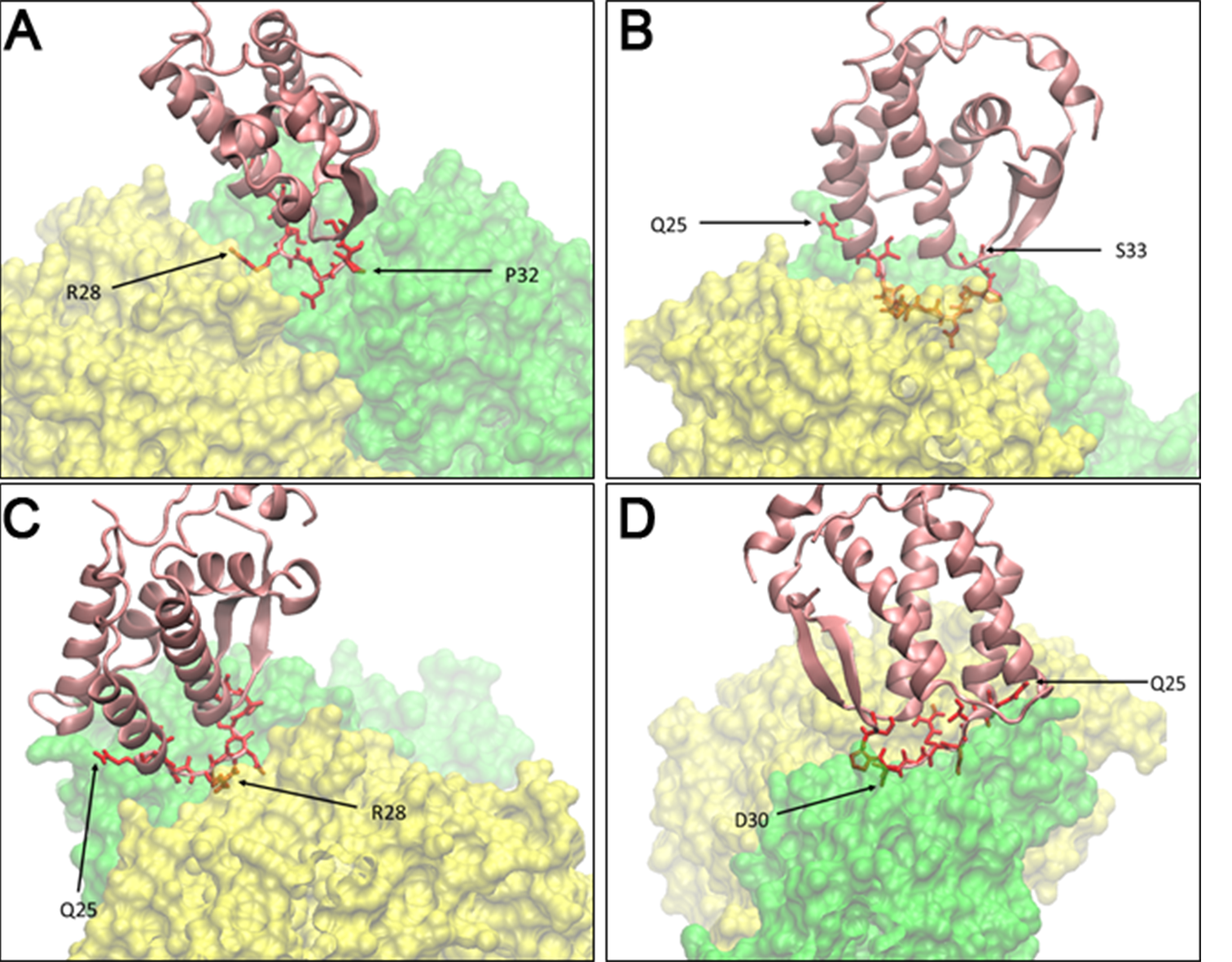

Supplement: S12 Fig — M-CSFRGD/αvβ3 integrin interface seen from different angles (A–D). αv in yellow “surf” presentation, β3 in green, M-CSFRGD in pink, and the mutant QTSRGDSPS loop in red. M-CSF, macrophage colony-stimulating factor; RGD, Arginine-Glycine-Aspartic acid. (TIF) [file pbio.2002979.s012.tif]

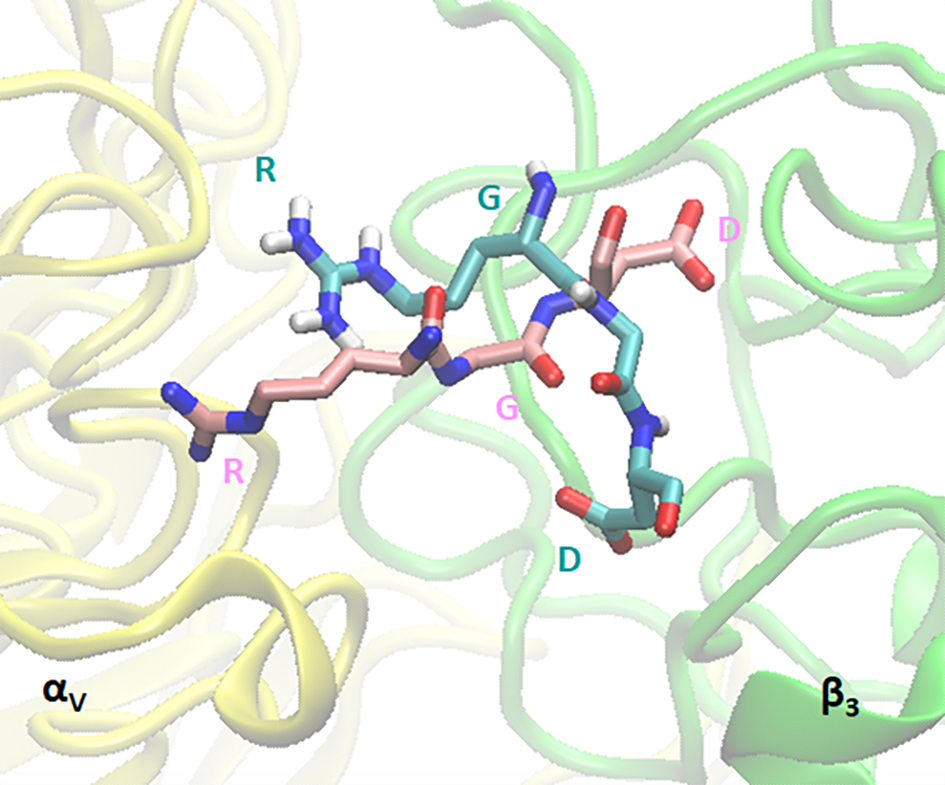

Supplement: S13 Fig — αv in yellow, β3 in green, RGD from M-CSFRGD in cyan, and RGD from the crystal in pink. cRGD, cyclic RGD; M-CSF, macrophage colony-stimulating factor; RGD, Arginine-Glycine-Aspartic acid. (TIF) [file pbio.2002979.s013.tif]

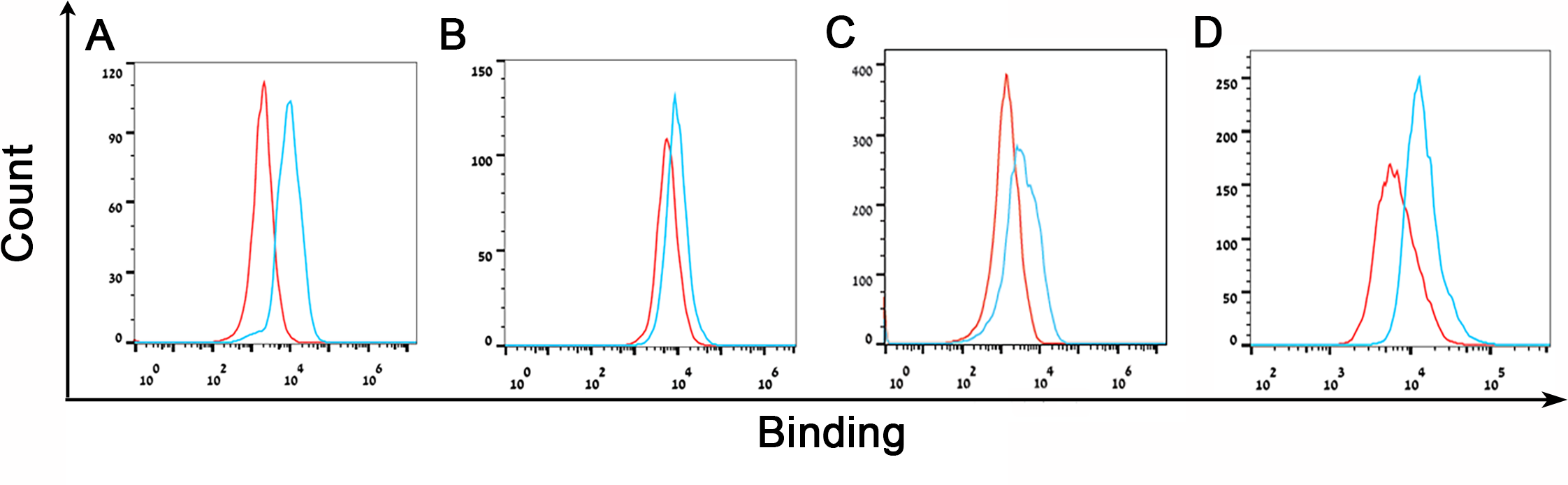

Supplement: S14 Fig — The expression levels of c-FMS and αvβ3 integrin were measured using flow cytometry. The red histograms represent the negative control, and the blue histograms represent receptor expression. Mouse BMMs without differentiation cytokines (t = 0) express c-FMS (A) and αvβ3 integrin (B). MDA-MB-231 breast cancer cell line express c-FMS (C) and αvβ3 integrin (D). BMM, bone-marrow–derived monocyte; MDA-MB-231, MDA Anderson metastatic breast 231. (TIF) [file pbio.2002979.s014.tif]

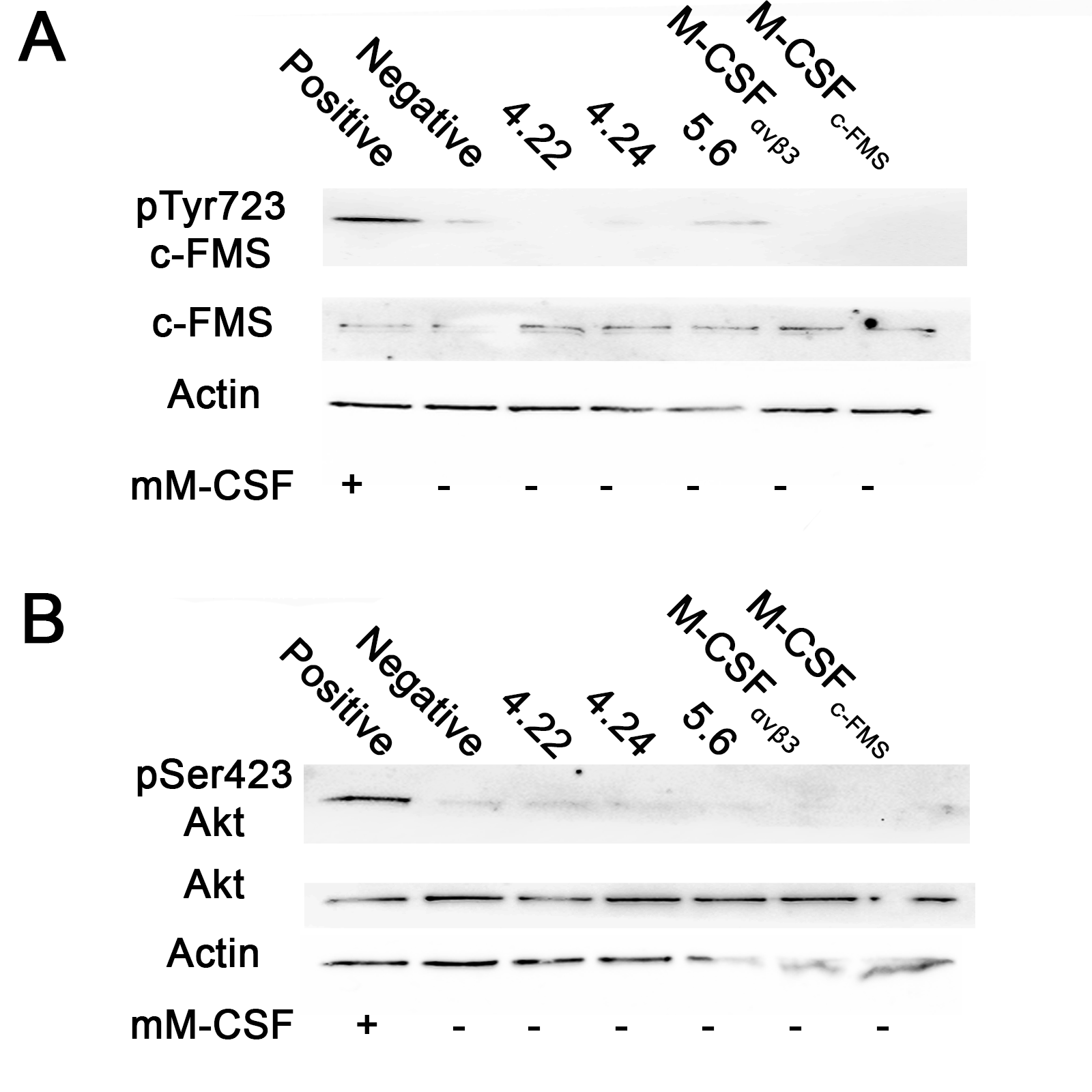

Supplement: S15 Fig — Murine BMMs were seeded for differentiation for 48 h, followed by incubation of purified M-CSFc-FMS, M-CSFαvβ3, and M-CSFRGD variants without the addition of murine M-CSF. Cells were lysed and subjected to SDS-PAGE to test spontaneous activation of (A) c-FMS and (B) Akt. The aspect ratios of the membranes were changed. BMM, bone-marrow–derived monocyte; M-CSF, macrophage colony-stimulating factor; RGD, Arginine-Glycine-Aspartic acid. (TIF) [file pbio.2002979.s015.tif]

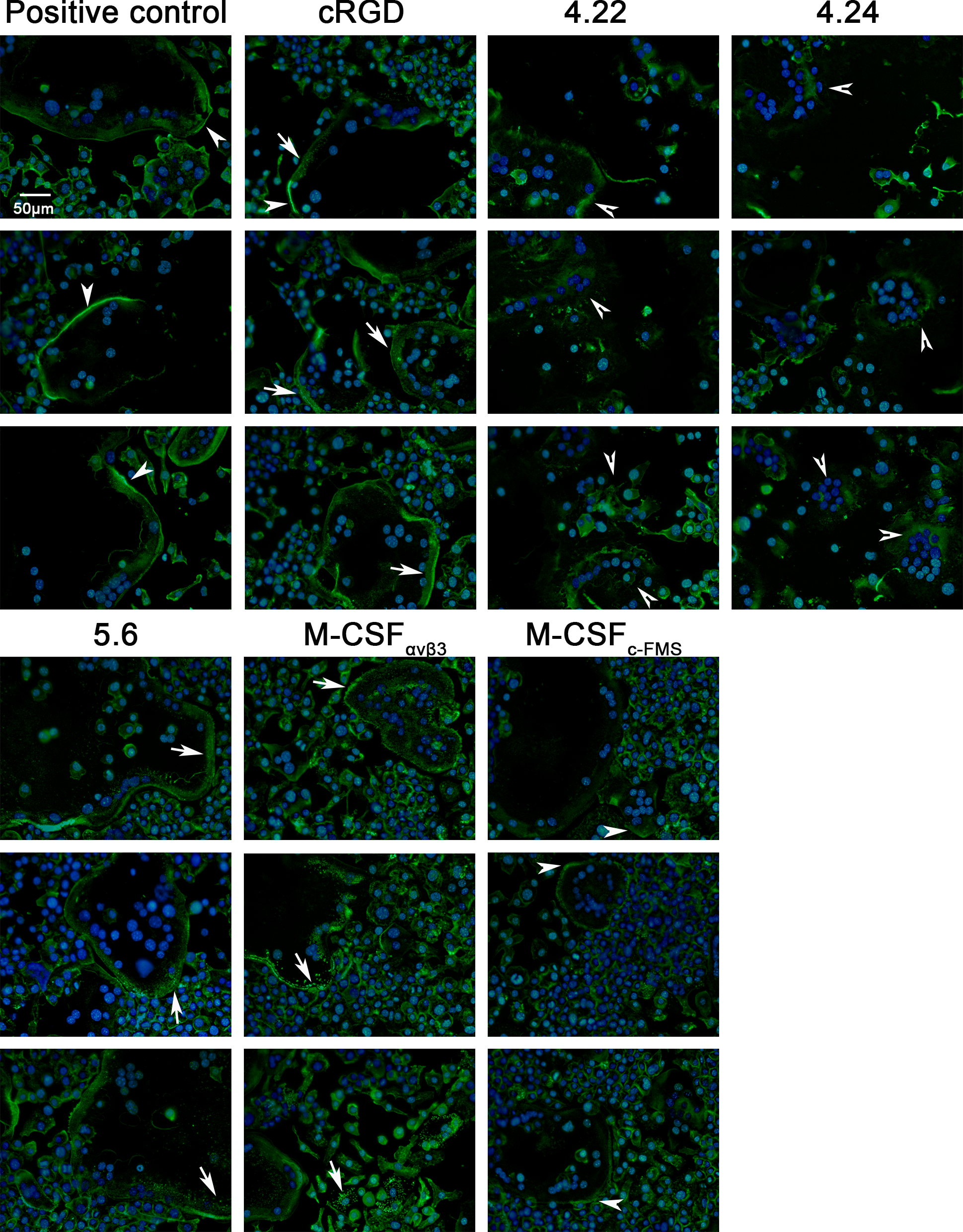

Supplement: S16 Fig — Differentiated murine BMMs were incubated for additional 24 h without (positive control) or with inhibitors (5 μM) followed by fixation and F-actin and nuclei staining. Cells were able to form a solid actin ring (white arrowheads), scattered actin ring [44] (white arrows) or amorphous actin distribution (barbed arrowheads). Pictures are representatives of 35 images acquired from five different wells per sample. BMM, bone-marrow–derived monocyte; M-CSF, macrophage colony-stimulating factor; RGD, Arginine-Glycine-Aspartic acid. (TIF) [file pbio.2002979.s016.tif]
