# Supplementary material for: A dual-specific macrophage colony-stimulating factor antagonist of c-FMS and αvβ3 integrin for osteoporosis therapy
Source: PLoS Biol. 2018 Aug 24;16(8):e2002979. doi: 10.1371/journal.pbio.2002979 (PMC6126843; doi:10.1371/journal.pbio.2002979)
Supplement: S1 Text — (DOCX) [file pbio.2002979.s017.docx]

**Supporting Information**

**Designing and constructing M-CSF_C31S_, two M-CSF_RGD_ libraries and M-CSF_αvβ3_**. Using the 3D structure of the murine c-FMS/M-CSF complex (PDB: 3EJJ) and the 3D structure of human M-CSF (PDB: 1HMC), the binding interface of the complex was identified. The M-CSF monomer, designated M-CSF_C31S_, which consists of 158 amino acids, was designed with a C31S mutation that prevents covalent (disulfide-linked) homodimerization of the monomer units [1]. Two libraries of M-CSF_C31S_ variants were then designed such that the two loops distant from the receptor binding site (residue numbering according to PDB: 3EJJ 25 to 32 and 64 to 71, respectively) were each replaced with an RGD motif flanked by three random amino acids on each side represented as XXXRGDXXX (designated M-CSF_RGD_). To generate the random amino acids in each library, the DNA codons were synthesized as NNS sequences, where N can be any codon and S can be C or G. The M-CSF_C31S_ gene and the two M-CSF_RGD_ libraries were prepared for us by GenScript (Piscataway, NJ, USA) with pCTCON homology sites and ECORI and NheI restriction sites on the amine terminal and AvrII and BamHI restriction sites on the C terminal. The M-CSF_C31S_ gene and M-CSF_RGD_ library DNA sequences were amplified by PCR with Phusion DNA polymerase (New England Biolabs, Ipswich, MA, USA) and transformed into *Saccharomyces cerevisiae* strain EBY100 with a modified linear pCTCON plasmid [2] for homologous recombination by using a Micropulser electroporator (Bio-Rad, USA). The DNA sequences were recovered in the tryptophan-selective medium SDCAA. To evaluate the diversities (sizes) of the two M-CSF_RGD_ libraries, the yeasts were plated on SDCAA plates (0.54% Na_2_HPO_4_, 0.856% Na_2_HPO_4_•H_2_O, 18.2% sorbitol, 1.5% agar, 2% dextrose, 0.67% yeast nitrogen base, 0.5% bacto casamino acids) at several dilutions and were counted manually. Library 1 consisted of 3.6×10^6^ variants, and library 2 of 8.3×10^6^ variants. Combining the two libraries into one resulted in 1.1×10^7^ variants. To evaluate the initial variability of the libraries, 20 clones from each library were sequenced by GenScript.

**Purification of proteins.** After identifying the best binders for c-FMS and α_v_β_3_ integrin, variant 4.22 was chosen to be mutated to become a binder of α_v_β_3_ integrin, but not of c-FMS; this mutant was designated M‑CSF_αvβ3_. Two single point mutations were designed in positions 9 and 15 by replacing histidine with alanine [3]. Point mutations were inserted into pPICK9K/M-CSF_RGD_ variant 4.22 to obtain the monospecific α_v_β_3_-integrin-binding clone (Biomatik, USA). To create a monospecific M-CSF that would bind c-FMS, but not α_v_β_3_ integrin, three single point mutations were introduced into M-CSF_RGD_ variant 4.22 by means of a polymerase chain reaction (PCR). The PCR was performed in a "round-the-horn" technique using Phusion High-Fidelity DNA Polymerase (New England Biolabs) and the phosphorylated primers RDG fwd 5'—3' and RDG rev 5'—3' (IDT, IA , USA). The three bispecific M-CSF_RGD_ variants (4.22, 4.24 and 5.6), M-CSF_c-FMS_ and M-CSF_αvβ3_ were digested with ECORI and AvrII (New England Biolabs) and ligated with Quick Ligase (New England Biolabs) to the pPICK9K expression plasmid containing a FLAG tag in the N-terminus and 6×His tag in the C-terminus of the cloning site. The plasmids were transformed into competent *E. coli* and sequenced with AOX1 forward and reverse primers as previously described. The plasmids with the desired sequence were linearized with SacI restriction enzyme (New England Biolabs) and transformed into *Pichia pastoris* strain GS115 by using the multi-copy Pichia Expression Kit (Invitrogen, USA). After transformation, the cells were plated on RDB plates (18.6% sorbitol, 2% agar, 2% dextrose, 1.34% yeast nitrogen base, 4×10–5% biotin and 5×10–3% each of l-glutamic acid, l-methionine, l-leucine, l-lysine, and l-isoleucine) for 48 h at 30°C, collected, and plated again on Geneticin (G418 4 mg/ml) plates for an additional 48–72 h. Colonies were transferred to 5 ml of BMGY medium (2% peptone, 1% yeast extract, 0.2 % K_2_H(PO_4_), 1.1812% KH_2_(PO_4_), 1.34% yeast nitrogen base, 4×10–5% biotin, 1% glycerol), incubated overnight at 30°C, and transformed into 5 ml of BMMY medium (2% peptone, 1% yeast extract, 0.23% K_2_H(PO_4_), 1.1812% KH_2_(PO_4_), 1.34% yeast nitrogen base, 4×10–5% biotin, 0.5% methanol) for 3 days at 30°C. Each day, methanol was added to the BMMY medium to maintain the concentration at 0.5%. For small-scale protein expression, the GS115 cells were centrifuged at 3800g for 10 min, and the supernatant was collected for western blot analysis using 1:1000 primary mouse anti-FLAG (Sigma-Aldrich), followed by 1:5000 anti-mouse secondary antibody conjugated to alkaline phosphatase (Jackson ImmunoResearch, West Grove, PA, USA), and 2 ml of BCIP reagent for signal development (Sigma-Aldrich). The highest expressing culture was grown in 50 ml of BMGY overnight at 30°C and transferred to 500 ml of BMMY. Each day (for 3 days), methanol was added to the BMMY medium to maintain the concentration at 0.5%. Then, cells were concentrated at 3800 rpm for 10 min, and the supernatant was filtered using 0.22 µm stericups (Millipore, Ecuador). NaCl was added to the filtrate to a final concentration of 150–300 mM, imidazole was added to a final concentration of 10 mM, and the pH was adjusted to 8.0. The protein solution was incubated for 1 h at 4 °C, centrifuged at 3800g for 10 min, and filtered again. The filtrate was loaded on a HisTrap Ni column (GE Healthcare Life Sciences, UK) using a peristaltic pump. The column was washed with binding buffer (20 mM NaH_2_PO_4_∙H_2_O, 500 mM NaCl, and 10 mM imidazole; pH 8) and eluted with elution buffer (20 mM NaH_2_PO_4_∙H_2_O, 500 mM NaCl, and 500 mM imidazole; pH 8). The elution buffer was removed by Vivaspin (GE Healthcare Life Sciences, UK) with a 3,000-Da cutoff, and the buffer was replaced with PBS. Protein N-glycosylations were removed by overnight treatment with the endoglycosidase Endo HF (New England Biolabs) at room temperature. Proteins were further purified on an ÄKTA Pure 150 FPLC with a Superdex 200 16/600 size exclusion column, and the elution times were compared to those for protein standards. Purified protein samples were subjected to mass spectrometry analysis (Ilse Katz Institute for Nanoscale Science and Technology, BGU) and to SDS-PAGE Coomassie blue staining with Instant Blue (Expedeon, San Diego, CA, USA) to evaluate protein purity. Protein concentrations were measured using an Evolution 260 bio spectrophotometer (Thermo Fisher Scientific, USA) based on protein absorption at 280 nm and an extinction coefficient of 13,325 M^-1^cm^-1^ for the M-CSF_RGD_ variant 4.22 and M-CSF_c-FMS_. The extinction coefficient for the M‑CSF_αvβ3_ and M-CSF_RGD_ variants 4.24 and 5.6 was 14,815 M^-1^cm^-1^.

**Circular dichroism (CD) analysis.** Secondary structural analysis of the purified proteins was performed using a J-815 CD spectrometer (JASCO, Tokyo, Japan) with a 1-mm path length quartz cuvette. Spectra of 5 μM of purified protein in 400 μl of PBS were obtained at room temperature. The average of three spectra was normalized to obtain ellipticity (degree × cm^2^/dmol) and the PBS background was subtracted. Data points with a diode voltage >1000 V were excluded. The melting temperature of each protein was determined at 217 nm. Measurements were performed in 1 ^0^C-gaps from 15 ^0^C to 95 ^0^C.

**Chemical cross linking.** Murine M-CSF_WT_, purified M-CSF_RGD_ variants, M-CSF_c-FMS_ and M-CSF_αvβ3_ were incubated with different concentrations of BS^3^ [bis(sulfosuccinimidyl)suberate, Thermo Fisher Scientific, Waltham, MA, USA] cross linker 0–2500 μM for 30 min at room temperature. Then, Tris was added to a final concentration of 30 mM, and the mixtures were incubated for 15 min at room temperature. Samples were denaturized and loaded on 15% SDS-PAGE. The gels were stained with Coomassie blue (InstantBlue, Expedeon) and visualized with MiniBis pro (DNR Bio-Imaging Systems, Jerusalem, Israel).

**References**

1. Deng, P., et al., *The role of individual cysteine residues in the processing, structure, and function of human macrophage colony-stimulating factor.* Biochemical and biophysical research communications, 1996. **228**(2): p. 557-566.

2. Rosenfeld, L., et al., *Combinatorial and Computational Approaches to Identify Interactions of Macrophage Colony-stimulating Factor (M-CSF) and Its Receptor c-FMS.* Journal of Biological Chemistry, 2015. **290**(43): p. 26180-26193.

3. Koths, K., *Structure‐function studies on human macrophage colony‐stimulating factor (M‐CSF).* Molecular reproduction and development, 1997. **46**(1): p. 31-38.
